# Secretory IgA Is a Key Marker Among Gut Barrier Dysfunction‐Related Immunoglobulins Predicting Outcomes in ACLF

**DOI:** 10.1111/liv.70350

**Published:** 2025-09-13

**Authors:** Boglarka Balogh, David Tornai, Aniko Csillag, Istvan Tornai, Zsuzsanna Vitalis, Patricia Kovats, Peter Antal‐Szalmas, Tamas Dinya, Wim Laleman, Minneke J. Coenraad, Jonel Trebicka, Maria Papp, MICROB‐PREDICT and PREDICT study group of the EASL‐CLIF consortium

**Affiliations:** ^1^ Division of Gastroenterology, Department of Internal Medicine Faculty of Medicine, University of Debrecen Debrecen Hungary; ^2^ Kalman Laki Doctoral School of Biomedical and Clinical Sciences, Faculty of Medicine University of Debrecen Debrecen Hungary; ^3^ Department of Laboratory Medicine, Faculty of Medicine University of Debrecen Debrecen Hungary; ^4^ Department of Surgery, Faculty of Medicine University of Debrecen Debrecen Hungary; ^5^ Department of Gastroenterology & Hepatology, Division of Liver & Biliopancreatic Disorders and Liver Transplantation University Hospitals Leuven, KU Leuven Leuven Belgium; ^6^ Department of Internal Medicine B University of Münster Münster Germany; ^7^ Department of Gastroenterology and Hepatology Leiden University Medical Center Leiden the Netherlands; ^8^ European Foundation for Study of Chronic Liver Failure Barcelona Spain

**Keywords:** acute‐on‐chronic liver failure, adaptive immunity, gut barrier failure, immunoglobulin A, secretory IgA, short‐term mortality

## Abstract

**Background and Aims:**

In cirrhosis, impaired gut mucosal immunity facilitates bacterial translocation (BT) instigating the proinflammatory cascade that exacerbates hepatic damage. The role of antibody‐mediated immunity in this process remains unclear. We assessed serum immunoglobulins (Ig) linked to gut barrier function as prognostic markers in a prospective MICROB‐PREDICT cohort of patients with acutely decompensated (AD) cirrhosis.

**Methods:**

Serum samples of 128 patients were assayed for IgA and IgG antibodies against various targets (filamentous‐actin; *Saccharomyces cerevisiae* [ASCA]; glycoprotein‐2 [GP2]; gliadin; endotoxin‐core [EndoCab]), secretory (s)IgA, total‐IgA, IgG, IgM and free Ig kappa/lambda light chains. Mortality was assessed during a 3‐month follow‐up period. An independent ACLF patient cohort (*n* = 50) was used to validate sIgA‐related findings.

**Results:**

IgA‐type target‐specific antibodies were more prevalent than IgG types. Target‐specific antibody diversity and concentrations, total‐IgA levels and Child–Pugh severity exhibited concordant elevations. Total‐IgG levels were inversely associated with CLIF‐C AD score and presence of ACLF. sIgA levels increased in parallel with ACLF grades. Elevated sIgA levels were associated with 90‐day mortality in ACLF patients (*n* = 37; AUROC: 0.859; at the cut‐off of > 20.9 μg/mL: 11.1% vs. 78.9% Mortality *p* < 0.001). These findings were confirmed in the validation cohort. In the merged ACLF cohort (*n* = 87), high sIgA levels predicted 90‐day mortality independent of CLIF‐C ACLF score (HR: 3.367; CI: 1.563–7.225; *p* = 0.002).

**Conclusion:**

Enhanced BT‐triggered immune activation is indicated by increased total‐IgA levels in association with the occurrence of target‐specific IgA antibodies. Serum sIgA is a promising marker of gut barrier failure and 90‐day mortality in ACLF.

**Summary**

- Liver cirrhosis can lead to damage to the gut's protective barrier, allowing harmful bacteria to enter the bloodstream and make patients sicker.
- In this study, we investigated different antibody levels in patients with severe liver disease during sudden health complications.
- The findings suggest that the immune system's antibody response becomes increasingly disturbed as the disease progresses.
- In the most severe patients, higher levels of a particular type of antibody called secretory IgA were linked to a greater chance of death within 3 months.
- This finding could help doctors identify patients at the highest risk.

**Abbreviations**
ACLF: acute‐on‐chronic liver failure
AD: acute decompensation
ASCA:
*Saccharomyces cerevisiae*

AUROC: area under the ROC curve
BT: bacterial translocation
CI: confidence interval
CLIF‐C: Chronic Liver Failure Consortium
EndoCab: endotoxin‐core antibody
F‐actin: filamentous‐actin
GP2: glycoprotein‐2
HR: hazard ratio
Ig: immunoglobulin
LC: light chains
ROC: receiver operating characteristics
SC: secretory component
sIgA: secretory IgA

## 1 Introduction

Gut‐liver interaction is a key component in the progression of chronic liver diseases. In patients with liver cirrhosis, the immunological barrier function of the gut mucosa may be disrupted, resulting in persistently increased leakage of bacterial antigens from the gut lumen to the portal circulation (i.e., bacterial translocation, BT). Enhanced BT triggers the activation of the proinflammatory signalling cascade that exacerbates tissue damage in both the gut and liver, consequently accelerating the progression of liver disease [1].

In the gut mucosa, the vast majority of B‐cells engage in immunoglobulin (Ig)A production. In normal circumstances, the secreted form of IgA antibodies (secretory IgA; sIgA) neutralise and mask bacterial antigens in the gut before they reach the epithelium, while they also shape the microbiota, providing protection and tolerance. sIgA is transported through the epithelial cells by the polymeric‐Ig receptor, a piece of which (secretory component; SC) remains bound to the antibody even after release to the intestinal lumen [2]. A retrograde (mucosal surface → systemic circulation) transportation mechanism of sIgA antibodies (by an unknown mechanism) has also been hypothesised [3, 4]. Notably, several types of cellular IgA receptors (Fcα/μ receptor, asialoglycoprotein receptor, transferrin receptor/CD71, SC receptor, M‐cell receptor and FcαRI/CD89) have been described [4] that might play a role in such reversed transport; however, passive leakage back to the circulation is also possible when permeability is severely increased.

Elevated circulating levels of total‐IgA, and most importantly also sIgA, have been reported in different chronic liver diseases [5, 6], which might indicate BT‐driven activation of B and plasma cells in the gastrointestinal tract and increased reuptake of sIgA or (possibly bacteria‐associated) sIgA back‐leakage.

In addition, several target‐specific IgA‐type antibodies have been shown to be associated with disease progression in liver and intestinal diseases. Our group has previously demonstrated that enhanced formation of IgA‐type antimicrobial (ASCA) and autoantibodies was associated with an increased development rate of systemic bacterial infection in cirrhosis. We also provided evidence for the gut mucosal immune system‐related origin of these antibodies by showing a marked increase in the proportion of IgA2 with increased presence of SC on these antibodies, linking them to gut‐driven antigens [7, 8].

IgA‐type autoantibodies against glycoprotein‐2 (GP‐2), a gut innate immunity protein, have been shown to be associated with a progressive disease course, such as the development of liver cirrhosis or cholangiocellular carcinoma in patients with primary sclerosing cholangitis (PSC) [3, 9, 10, 11]. GP‐2 can interact with FimH‐positive bacteria bearing type‐1 fimbriated pyli and act as a microbiome‐sensing receptor while maintaining the equilibrium of the gut microbiota [12].

Anti‐F‐actin IgA (directed against cytoskeletal actin filaments) and anti‐gliadin IgA have also been linked to structural damage to the intestinal mucosa. The presence of anti‐F‐actin IgA was reported to strongly correlate with the histological degree of small intestinal atrophy in patients with celiac disease [13] and could identify PSC patients with a progressive disease course and poorer survival [14]. The presence of anti‐gliadin IgA is associated with increased intestinal permeability and significant portal hypertension in patients with cirrhosis [15].

In response to LPS exposure, so‐called endotoxin core antibodies (EndoCabs) are produced. The antigen for these antibodies is the hydrophobic lipid A, which forms the inner core of lipopolysaccharides (LPS) and is responsible for the majority of endotoxin toxicity. Lipid A displays little strain variation among different bacteria [16]. Therefore, EndoCab IgA was hypothesised to be a marker of host response to intestinal BT.

The light chain (LC) of human Ig is of two types: kappa and lambda. Elevated free IgLC levels have been observed in the serum and tissue of patients with inflammatory bowel disease (IBD) and have been reported to play a role in mast cell degranulation [17].

However, little is known about the role of these markers of mucosal immune activation in the context of acute decompensation (AD) and acute‐on‐chronic liver failure (ACLF) in patients with cirrhosis. Therefore, we measured IgA and IgG isotypes of the above‐mentioned target‐specific antibodies along with total‐IgA, IgG, IgM, sIgA, free kappa and lambda IgLC levels in a prospective cohort of patients with cirrhosis and AD or ACLF and investigated their association with disease severity and outcomes.

## 2 Patients and Methods

### 2.1 Patients

The discovery cohort comprised 130 patients; however, two patients with IgA deficiency were excluded. The final cohort included 128 patients hospitalised for AD cirrhosis with (*n* = 20) or without (*n* = 108) ACLF. These patients were recruited in the frame of the MUCOSA‐PREDICT project between March 2017 and July 2018 exclusively from the Division of Gastroenterology, Department of Internal Medicine of the University of Debrecen and followed prospectively.

Patients with AD were categorised according to high (≥ 50) or low CLIF‐C AD score [18] at inclusion and according to the 3‐month outcome at the end of the follow‐up period, as described by the PREDICT study previously (i.e., stable AD (SDC), unstable AD (UDC), pre‐ACLF) [19]. Patients with ACLF were identified according to the previously established criteria [20]. The study protocol has been previously published [19]. Clinical information was collected at baseline and scheduled follow‐up visits at weeks 1, 4, 8 and 12. In addition, unscheduled visits were performed during the course of the study at the time of hospital readmission due to a new AD episode or ACLF development. The latter was followed up with another visit 1 week after the development of ACLF. Of the 22 non‐ACLF patients who progressed to ACLF within 90 days of inclusion (i.e., pre‐ACLF) serum samples were obtained from 17 patients at the time of ACLF onset. At the end of the follow‐up period, clinical data were exported from the study database, including medical history, clinical and laboratory results and outcomes.

Additionally, 50 patients with ACLF were also enrolled in the University of Debrecen from the period between December 2014 and February 2017. Serum samples of these patients were exclusively used to validate the 90‐day mortality‐predicting ability of sIgA in ACLF patients.

The study protocol was approved by the Regional and Institutional Research Ethics Committee of the University of Debrecen and the National Scientific and Research Ethics Committee (4722−/2014/EKU, 9485–1/2016/EKU, 58361–2/2016/EKU, 35281–2/217/EKU and 41 192–5/2018/EÜIG) and within the framework of the PREDICT study. Each patient or legal representative was informed of the nature of this study and signed an informed consent form concerning participation in the study and the publication of the collected data.

### 2.2 Samples and Evaluation of Serological Biomarkers

Blood samples were collected from each patient at enrolment and at the time of readmission due to ACLF development. The sera were isolated after half an hour and kept frozen at −80°C until testing. All serological assays were performed in a blinded fashion without prior knowledge of the patient's clinical information. Commercially available ELISA kits were applied according to the manufacturer's instructions to determine the concentration of various general mucosal immune activation‐related and target‐specific IgA and IgG type anti‐microbial and auto‐antibodies according to the following: Secretory IgA (Abnova; Taipei, Taiwan; cat. #: KA3980); Free kappa and lambda IgLC (Biovendor; Brno, Czech Republic; cat. #: RD194088100R); EndoCab IgA (Hycult Biotech; Uden, Netherlands; cat. #: HK504‐IgA); Anti‐Gliadin IgA and IgG (INOVA Diagnostics; San Diego, CA, USA; cat. #: 708 655 and 708 650); Anti‐F‐actin IgA and IgG (INOVA Diagnostics; San Diego, CA, USA; cat. #: 704 500 and 708 785); ASCA IgA and IgG (GA Generic Assays GmbH; Dahlewitz, Germany; cat. #: 4006 and 4007); Anti‐GP2 IgA and IgG (GA Generic Assays GmbH; Dahlewitz, Germany; cat. #: 3750 and 3850). sCD163 and sCD206 levels were determined by commercially available solid‐phase enzyme‐linked immunoassays (IQProducts, Groningen, Netherlands, Cat.: IQP‐383; and Ray Biotech, Norcross, GA, USA, Cat.: ELH‐MMR respectively) using an ETI‐MAX 3000 machine. Presepsin levels were measured by a chemiluminescent PATHFAST presepsin analyser (Mitsubishi Chemical Medience Corporation, Tokyo, Japan). Total‐IgA, IgG and IgM levels were measured by turbidimetry in the routine laboratory unit of the University of Debrecen.

### 2.3 Statistical Analysis

Variables were tested for normality using the Shapiro–Wilk W test. Categorical variables were summarised as frequencies and percentages and compared with χ2 test. Continuous variables were summarised as medians and interquartile range (IQR, lowest 25%‐highest 25%) and were compared with the Mann–Whitney U test or Kruskal–Wallis H‐test with Dunn's multiple comparison post hoc analysis. The Spearman's nonparametric rank correlation test was used to determine correlations. The ability of different continuous variables to discriminate between survivors and nonsurvivors was assessed by receiver operating characteristic (ROC) curve analysis, plotting sensitivity% versus 100‐specificity%. The area under the curve (AUROC) and the corresponding 95% confidence intervals (CI) were calculated. The Youden index, indicating the maximum value of sensitivity + specificity, was chosen to estimate the best discriminative threshold. Kaplan–Meier survival curves were plotted to estimate the cumulative probability of 90‐day survival in the investigated groups. Differences in observed survival rates were assessed using the log‐rank test. The association between sIgA and mortality during the follow‐up was evaluated by univariable Cox‐regression analysis. Multivariable analysis was performed to adjust for CLIF‐C ACLF score or ACLF stage with the forced entry method. Associations are given as hazard ratios [HR] with 95% confidence intervals [CI]. For statistical analysis and graphical presentation, the SPSS v.29.0 (SPSS, Chicago, IL) and GraphPad Prism 10.2.1 (San Diego, CA) programs were used. A two‐sided probability value of < 0.05 was considered to be statistically significant.

## 3 Results

### 3.1 Baseline Data and Frequency of Disease‐Related Target‐Specific Antibodies

Clinical and laboratory data of patients with AD cirrhosis of the discovery cohort, as well as 28‐ and 90‐day mortality data, are summarised in Table 1. The frequencies of disease‐related target‐specific antibodies are also indicated. IgA‐type antibodies were generally more frequent than IgG types (*p* < 0.001 for all).

**TABLE 1:** Clinical and laboratory data of patients with acutely decompensated cirrhosis (*n* = 128).

Age	61 (53–68)
Sex (m/f)	84/44 (65.6%/34.4%)
Aetiology (alcohol/viral/both/other)	107/5/5/11 (83.6%/3.9%/3.9%/8.6%)
SDC/UDC/pre‐ACLF/ACLF	68/18/22/20 (53.1%/14.1%/17.2%/15.6%)
CLIF‐C AD score < 50/≥ 50	39/69 (30.5% / 53.9%)
CLIF‐C AD score	52 (47–59)
CLIF‐C ACLF score	49 (43–51)
MELD score	16 (11–21)
Child–Pugh stage A/B/C	17/56/55 (13.3%/43.7%/43%)
White blood cells	7.61 (5.2–10.2)
Platelets	126 (85–186)
INR	1.35 (1.13–1.64)
Albumin (g/L)	30 (25–35)
AST (U/L)	73 (35.8–147.3)
ALT (U/L)	32 (19–54)
ALP (U/L)	155 (100–225)
γGT (U/L)	214 (74.5–471)
Bilirubin (μmol/L)	60.2 (18.6–143.4)
Creatinine (μmol/L)	78 (52–108)
CRP (mg/L)	26.9 (9.9–52.1)
Ascites	92 (71.9%)
Hepatic encephalopathy	32 (25.0%)
Gastrointestinal bleeding	30 (23.4%)
ASCA IgA	62 (48.4%)
ASCA IgG	23 (18.0%)
Anti‐F‐Actin IgA	84 (65.6%)
Anti‐F‐Actin IgG	47 (36.7%)
anti‐gliadin IgA	72 (56.3%)
anti‐gliadin IgG	20 (15.6%)
anti‐GP2 IgA	31 (24.2%)
anti‐GP2 IgG	9 (7.0%)
mortality (28‐day)	13 (10.2%)
mortality (90‐day)	26 (20.3%)

*Note:* Data are presented as median (interquartile range) or *n* (%). Fischer's exact test for differences between IgA and IgG type antibodies resulted in *p* < 0.001 for all pairs.

Abbreviations: γGT, gamma‐glutamyl transferase; ACLF, acute‐on‐chronic liver failure; AD, acute decompensation; ALP, alkaline phosphatase; ALT, alanine aminotransferase; ASCA, anti‐Saccharomyces cerevisiae antibody; AST, aspartate aminotransferase; CLIF‐C, chronic‐liver failure consortium; CRP, C‐reactive protein; GP2, pancreatic glycoprotein 2; Ig, immunoglobulin; INR, international normalised ratio; MELD, model for end‐stage liver disease; SDC, stable decompensated cirrhosis; UDC, unstable decompensated cirrhosis.

Correlation analyses between measured markers, routine laboratory parameters and clinical scores are presented in Table S1–S4. Association between alcoholic aetiology and markers is presented in Table S5. Total IgA, sIgA and ASCA IgA antibodies demonstrated increased levels in patients with alcoholic aetiology (*p* < 0.001, =0.009, 0.010 respectively). ASCA IgA antibodies also showed increased frequency (53.6% vs. 12.5%, *p* = 0.002) in patients with alcoholic aetiology (Table S6).

### 3.2 Excessive Activation of the Adaptive Immune System Leads to the Formation of Disease‐Related Target‐Specific Antibodies

**FIGURE 1:**
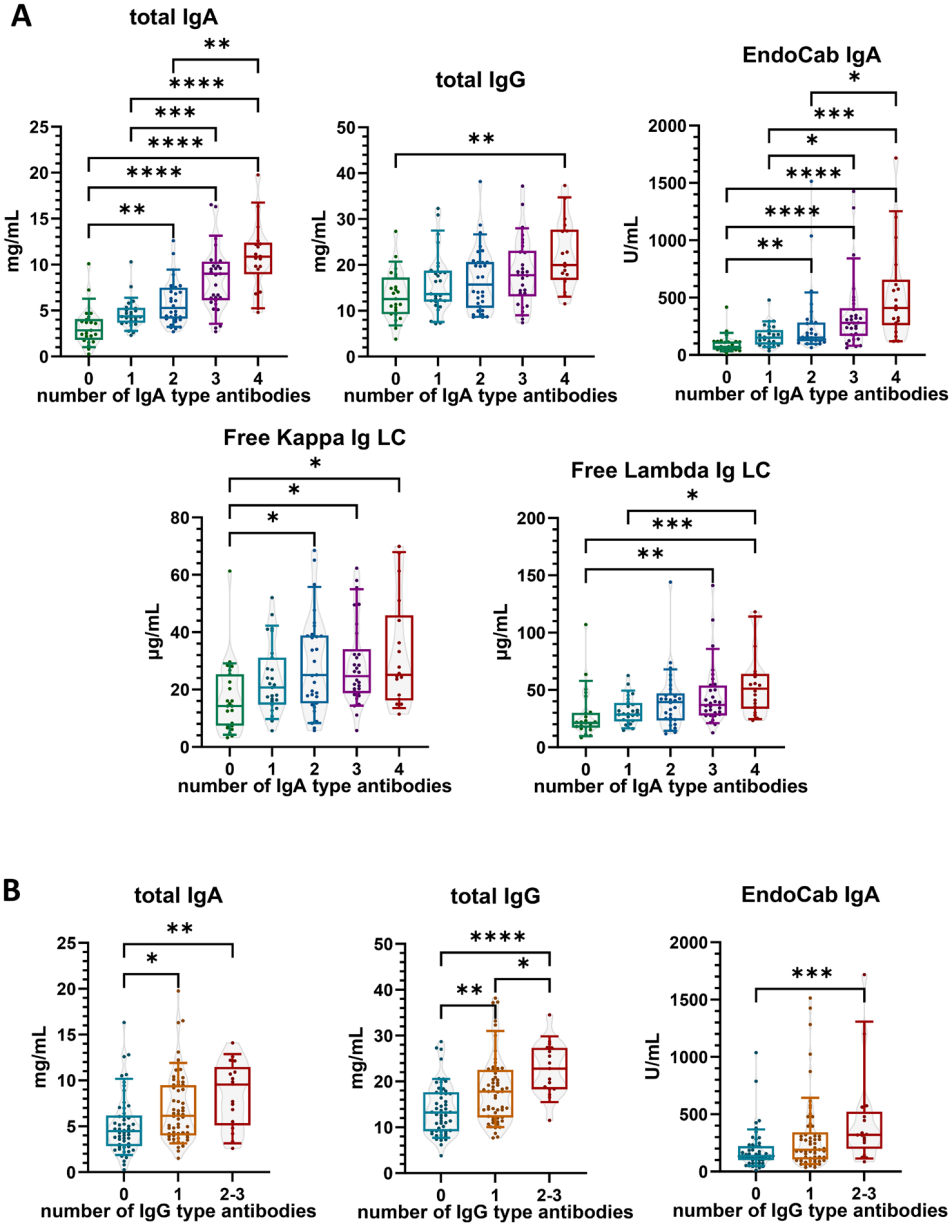
General activation of the adaptive immune system is associated with increased formation of disease‐related target‐specific antibodies. Occurrence of IgA‐type disease‐related target‐specific antibodies (A) showed a stronger association with the level of total‐IgA, while the number of IgG‐type disease‐related target‐specific antibodies (B) showed a stronger association with the level of total‐IgG. Since IgG‐type disease‐related target‐specific antibodies were less frequent, no patient in our cohort had all four investigated disease‐related target‐specific IgG antibodies. Furthermore, the combination of groups including patients with two or three types of IgG antibodies was necessary due to low numbers in individual groups. Ig: Immunoglobulin; LC: Light chain. Bars‐and‐whiskers indicate 10–25–50‐75‐90% of the values. * indicates *p* < 0.05; ** indicates *p* < 0.01; *** indicates *p* < 0.001; **** indicates *p* < 0.0001. Where significance level is not indicated: *p*> 0.05.

The higher the total‐IgA and IgG levels, the more types of the measured disease‐related target‐specific IgA and IgG antibodies were present in patients. A similar association was observed between increased levels of EndoCab IgA and the number of both IgA and IgG type antibodies (Figure 1A,B and Table S7). Free kappa and lambda IgLC levels were only associated with the number of IgA‐type antibodies in Kruskal–Wallis analysis (Figure 1A), a weak positive correlation was detected between IgLCs and the number of IgG‐type antibodies as well (Table S7). Furthermore, increased levels of total‐IgA and IgG were correlated with the levels of individual disease‐related target‐specific IgA antibodies (Table S1).

### 3.3 Disease Severity Is Associated With Immunoglobulin Production

**FIGURE 2:**
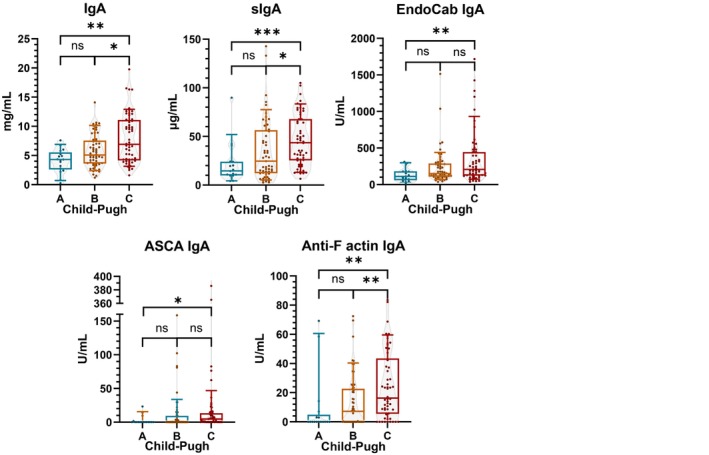
Child–Pugh stages were associated with increased levels of different IgA parameters. ASCA: Anti‐saccharomyces cerevisiae antibody; Ig: Immunoglobulin; sIgA: Secretory IgA. Bars‐and‐whiskers indicate 10–25–50‐75‐90% of the values. * indicates *p* < 0.05; ** indicates *p* < 0.01; *** indicates *p* < 0.001; ns indicates *p* > 0.05.

Of the severity scores, more advanced Child–Pugh stages were associated with an increased number of IgA‐ (Table 2) but not IgG‐type (data not shown) disease‐related target‐specific antibodies (χ^2^‐test *p* = 0.004). Similarly, Child–Pugh stages were associated with elevated levels of total‐IgA, sIgA, EndoCab IgA, anti‐F‐actin IgA and ASCA IgA (Figure 2). A positive correlation was also detected between these antibodies and Child–Pugh scores (Table S8). Additionally, the MELD score was correlated with sIgA (*r* = 0.383, *p* < 0.001) and anti‐F actin‐IgA (r = 0.200, *p* = 0.024) levels.

**TABLE 2:** Increased occurrence of IgA‐type disease‐related target‐specific antibodies is associated with more advanced Child–Pugh stages—Cross‐table analysis.

		Number of IgA‐type antibodies				
		0	1	2	3	4
Child–Pugh	A	5 (21.7%)	6 (22.2%)	5 (17.2%)	1 (3.3%)	0 (0.0%)
	B	10 (43.5%)	17 (63.0%)	13 (43.3%)	11 (36.7%)	5 (27.8%)
	C	8 (34.8%)	4 (14.8%)	12 (40.0%)	18 (60.0%)	13 (72.2%)

*Note:* χ^2^‐test *p* = 0.004.

Patients with ACLF had increased free kappa IgLC levels compared to AD patients without ACLF at inclusion (median [IQR]: 29.6 [20.18–40.65] vs. 20.5 [14.23–31.93] μg/mL; *p* = 0.026). AD patients without ACLF and with CLIF‐C AD score of ≥ 50 had decreased total‐IgG level compared to those with CLIF‐C AD score < 50 (16.00 [11.15–19.89] vs. 18.80 [13.45–24.05] mg/mL; *p* = 0.042).

**FIGURE 3:**
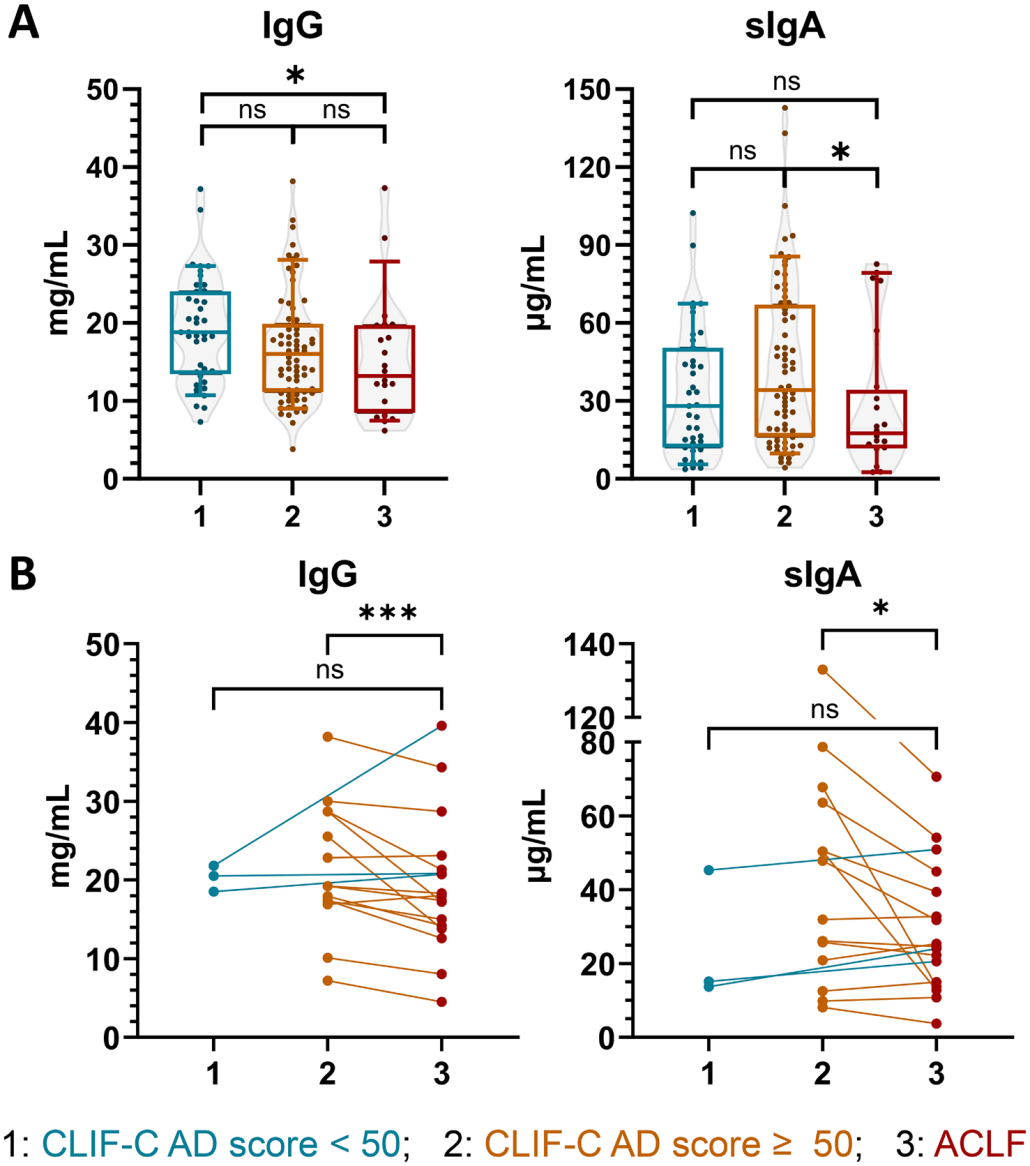
Total‐IgG and sIgA levels are associated with severity groups of acute decompensation, both cross‐sectionally (Part A) and longitudinally (Part B). Ig: Immunoglobulin; sIgA: Secretory IgA. Bars‐and‐whiskers indicate 10–25–50‐75‐90% of the values. *** indicates *p* < 0.001; * indicates *p* < 0.05; ns indicates *p* > 0.05.

Comparing all three groups, we observed a statistically significant decreasing trend in total‐IgG levels (CLIF‐C AD score < 50 vs. ≥ 50 vs. ACLF). There was a nonsignificant increase in sIgA levels according to severity within AD patients without ACLF. However, ACLF patients had significantly decreased sIgA levels compared to patients with CLIF‐C AD score ≥ 50 (Figure 3A). Significant decreases in total‐IgG and sIgA levels at the time of ACLF development compared to admission values were confirmed longitudinally in patients admitted as pre‐ACLF with CLIF‐C AD score ≥ 50 (Figure 3B).

### 3.4 Markers Are Associated With 28‐ and 90‐Day Adverse Outcomes

ACLF development within 90 days was associated with increased free Ig kappa light chain levels (median [IQR]: 28.45 [17,68‐39,58] vs. 18.85 [13.63–28.25] μg/mL; AUROC [95% CI]: 0.663 [0.537–0.790] *p* = 0.018).

Twenty‐eight‐day mortality was associated with increased total‐IgA to total‐IgG ratio (median [IQR]: 0.36 [0.26–0.49] vs. 0.53 [0.33–0.68]; AUROC [95% CI]: 0.683 [0.513–0.853], *p* = 0.031).

**FIGURE 4:**
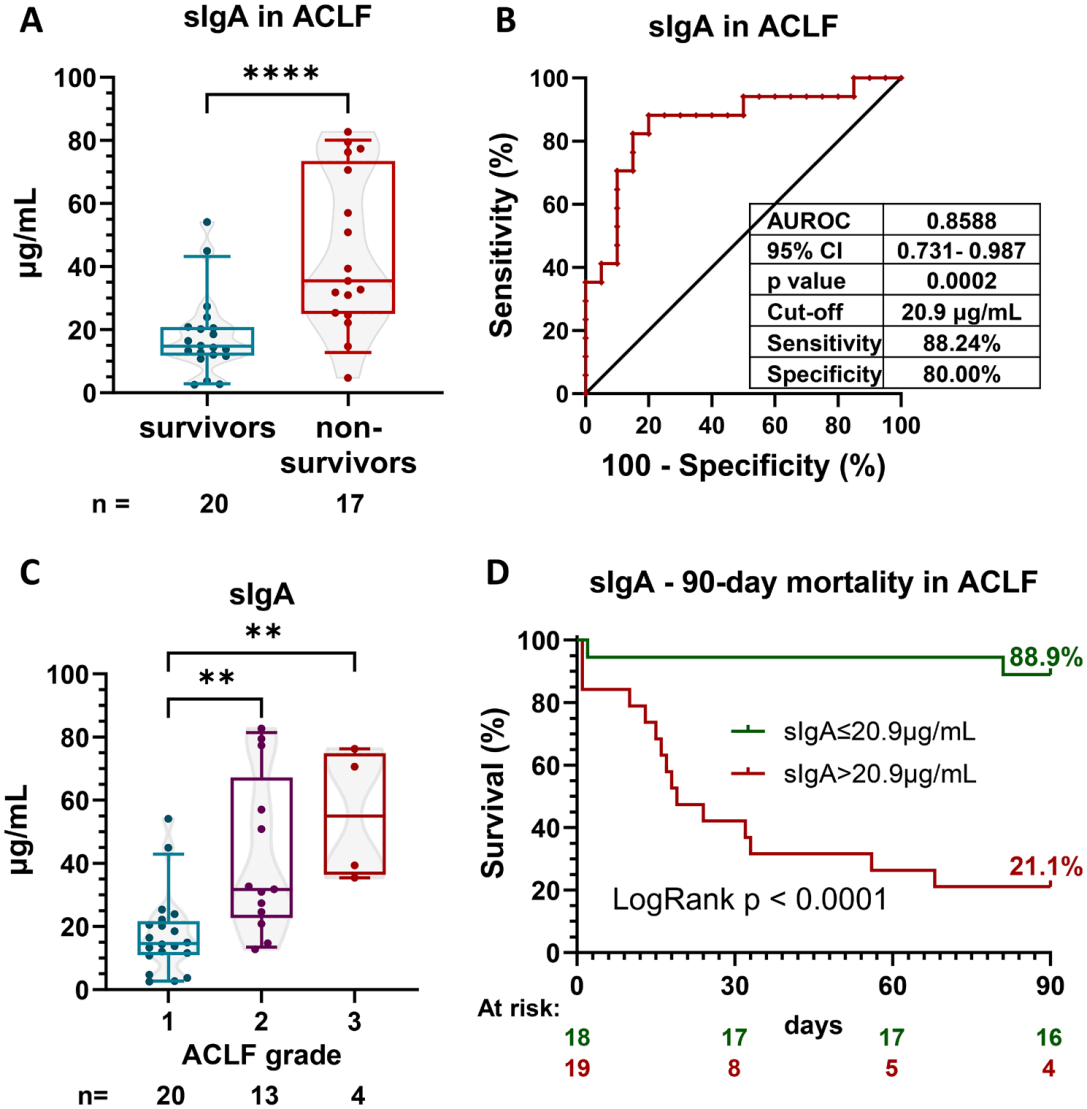
Serum secretory immunoglobulin A (sIgA) is associated with severity and 90‐day mortality in patients with acute‐on‐chronic liver failure (ACLF). Serum sIgA levels are significantly elevated in nonsurvivor patients compared to survivors (A) and increased in parallel with ACLF grades (C). In receiver operating characteristic (ROC) analysis, increased sIgA levels could discriminate between survivors and nonsurvivors. The best discriminatory cut‐off level is defined (B). Based on this cut‐off, increased sIgA levels are associated with increased incidence of 90‐day mortality in Kaplan–Meier analysis. Numbers of patients at risk are displayed every 30 days (D). Bars‐and‐whiskers indicate 10–25–50‐75‐90% of the values. ** indicates *p* < 0.01; **** indicates *p* < 0.0001. Where significance level is not indicated: *p*> 0.05.

However, 90‐day mortality was not associated with any of the measured markers in the whole patient population. On the other hand, in ACLF patients, including data and samples at the time of ACLF development of patients originally enrolled as pre‐ACLF (*n* = 37, mortality: 45.9%), sIgA was significantly higher in nonsurvivor patients compared to survivors (median [IQR]: 35.45 [24.99–73.46] vs. 14.67 [11.71–20.81] μg/mL) with a great AUROC (0.859, *p* < 0.001; Figure 4A–C). Additionally, we observed a gradual increase in serum sIgA levels according to ACLF grade (Figure 4C) and a positive correlation with CLIF‐C ACLF score (*r* = 0.499, *p* = 0.002). Kaplan–Meier curve, based on the best discriminatory cut‐off value (> 20.90 μg/mL; associated sensitivity: 88.2% and specificity: 80.0%), showed a higher mortality rate in patients with high sIgA levels compared to patients with low levels (78.9% vs. 11.1% LogRank *p* < 0.001; Figure 4D). In Cox regression, both logarithmically transformed (HR: 3.200; 95% CI: 1.584–6.465; *p* = 0.001) and dichotomised sIgA levels (HR: 12.951; 95% CI: 2.905–57.732; *p* < 0.001) were significant predictors of 90‐day mortality in ACLF patients.

**FIGURE 5:**
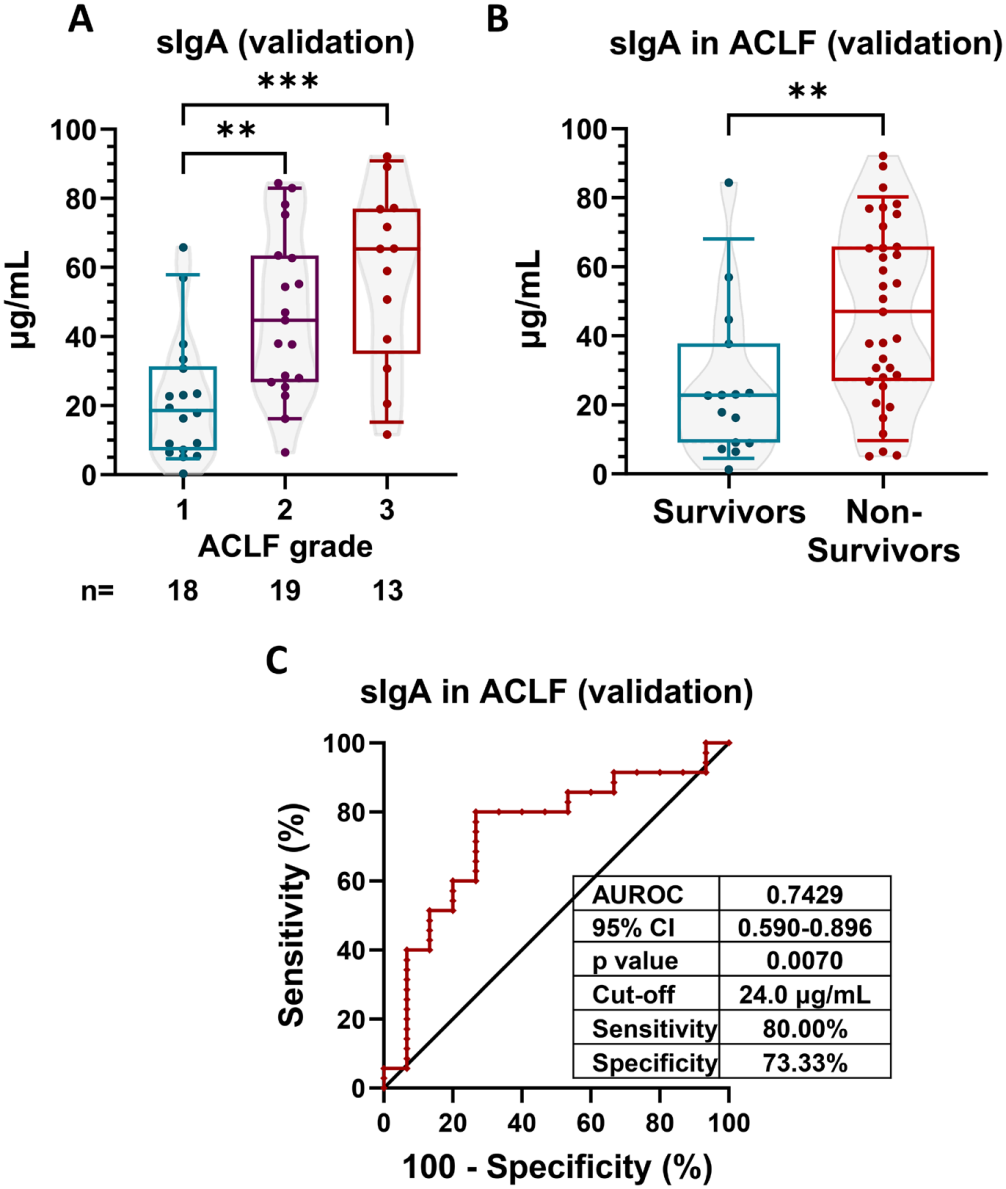
Serum secretory immunoglobulin A (sIgA) is associated with severity and 90‐day mortality in the validation cohort of patients with acute‐on‐chronic liver failure (ACLF). Serum sIgA levels are increased in parallel with ACLF grades (A) and are significantly elevated in nonsurvivor patients compared to survivors (B). In receiver operating characteristic (ROC) analysis, increased sIgA levels could discriminate between survivors and nonsurvivors. The best discriminatory cut‐off level is defined (C). Based on this cut‐off, increased sIgA levels are associated with increased incidence of 90‐day mortality in Kaplan–Meier analysis. Numbers of patients at risk are displayed every 30 days (D). Bars‐and‐whiskers indicate 10–25–50‐75‐90% of the values. **indicates *p* < 0.01; ***indicates *p* < 0.001. Where significance level is not indicated: *p* > 0.05.

In the validation cohort (*n* = 50; patient characteristics are summarised in Table S9), we found a higher mortality ratio (70%) that was most probably due to a higher number of more severe ACLF grades compared to the discovery (PREDICT) cohort (Figures 4C and 5A). Regardless of these differences, increased sIgA concentration was associated with higher ACLF categories (Figure 5A), correlated with CLIF‐C ACLF score (*r* = 0.517, *p* < 0.001) and nonsurvivors had significantly increased sIgA levels (Figure 5B; median [IQR]: 46.93 [26.80–65.83] vs. 22.70 [8.99–37.74] μg/mL) also in the validation cohort. The associated AUROC was 0.743 (*p* = 0.007) with a best discriminatory cut‐off value of > 24.0 μg/mL (sensitivity: 80.0% and specificity: 73.3%; Figure 5C). In Cox regression, logarithmically transformed (HR: 1.77; 95% CI: 1.152–2.720; *p* = 0.009) and dichotomised sIgA levels by the best discriminatory cut‐off level of both the discovery (HR: 2.471; 95% CI: 1.067–5.725; *p* = 0.035) and the validation cohort (HR: 3.912; 95% CI: 1.683–9.095; *p* = 0.002) all confirmed that sIgA is a significant predictor of 90‐day mortality in ACLF patients.

To correct for the severity of ACLF, we combined the two cohorts, now including a total of 87 ACLF patients. In multivariable Cox regression, logarithmically transformed serum sIgA concentration was not, but a high serum sIgA level according to either cut‐off value was found to be a predictor of 90‐day mortality independent of CLIF‐C ACLF score (Table 3). Similar results were obtained correcting for ACLF grade instead of CLIF‐C ACLF score (Table S10). Additionally, associations were assessed between sIgA levels and individual organ failures in ACLF patients. We observed increased sIgA levels in patients experiencing liver, cerebral, circulatory and respiratory failures, while renal failure was associated with lower sIgA levels (Table S11).

**TABLE 3:** High serum sIgA levels according to both discovery and validation cohort cut‐offs predict 90‐day mortality independently of CLIF‐C ACLF score in the merged ACLF cohort (*n* = 87).

ACLF patients (merged cohort) 90‐day mortality	Multivariable Cox regression analysis		
	HR	95% CI	*p*
CLIF‐C ACLF score	1.086	1.050–1.123	< 0.001
(Ln)sIgA	1.465	0.984–2.181	0.060
CLIF‐C ACLF score	1.085	1.051–1.120	< 0.001
sIgA (> 20.9 μg/mL)	2.812	1.299–6.086	0.009
CLIF‐C ACLF score	1.077	1.041–1.113	< 0.001
sIgA (> 24.0 μg/mL)	3.367	1.563–7.225	0.002

Abbreviations: ACLF, acute‐on‐chronic liver failure; CI, confidence interval; HR, hazard ratio; Ln, logarithmically transformed; sIgA, secretory immunoglobulin A.

Finally, as nonselective beta‐blocker (NSBB) therapy was proposed to have a stabilising effect on gut barrier function [15, 21], a sensitivity analysis was conducted to evaluate the potential confounding effect of NSBBs on the prognostic utility of serum sIgA. This analysis was performed in the discovery cohort, where data on medication were collected (11 treated and 26 nontreated patients). Initial analysis revealed no significant difference in serum sIgA concentrations between patients receiving NSBB therapy and those not receiving such treatment (median [IQR]: 22.24 [13.80–39.40] vs. 22.77 [12.94–42.26] μg/mL, *p* = 0.961). Subsequently, mortality rates were compared between treatment groups. Although the proportion of deceased patients was numerically lower in the NSBB‐treated group compared to the untreated group (36.4% vs. 50.0%), this difference did not achieve statistical significance in this limited population size (Table S12). However, analysis of sIgA's discriminative performance for 90‐day mortality revealed a marked disparity between treatment groups (Table S13). In patients not treated with NSBBs, serum sIgA demonstrated excellent prognostic performance for 90‐day mortality (AUROC: 0.959, *p* < 0.001). In stark contrast, in patients who were treated with NSBBs, the prognostic ability of sIgA was substantially attenuated (AUROC = 0.571, *p* = 0.705). Consistently, NSBB‐untreated patients exhibited distinct sIgA level distributions according to survival status, while in NSBB‐treated patients, median sIgA concentrations remained similar between survivors and nonsurvivors, clustering near the overall cohort median. These findings support the proposed intestinal barrier function‐stabilising effect of NSBB therapy, by which NSBB therapy seems to modulate the relationship between elevated serum sIgA concentrations and short‐term mortality.

## 4 Discussion

In the present study, we investigated the association between markers of intestinal immune function and disease severity as well as short‐term mortality in patients with AD cirrhosis.

The importance of gut‐liver interplay in the progression of cirrhosis has been widely recognised [1] contributing to immune dysfunction [22, 23] with susceptibility to infections [24, 25, 26, 27], development of multi‐organ dysfunction [20, 28, 29] and mortality [30, 31, 32, 33]. IgA, as the main immunoglobulin in the gut, is particularly affected by BT‐triggered immune activation. Previous studies have reported elevated total‐IgA levels in cirrhosis [6]. Furthermore, the emergence of disease‐related target‐specific antimicrobial and autoantibodies has been observed in various intestinal and liver diseases [3, 7, 8, 13, 34]. We propose that these changes are interconnected and influenced by BT. We hypothesise that BT‐induced excessive stimulation of IgA production ultimately contributes to the development of distinct autoreactive IgA subtypes in patients with cirrhosis. Indeed, parallel with elevated IgA levels, an increasing variety of disease‐related target‐specific antibodies were detected in patients' sera. Since BT activates the immune system as a whole, similar, although weaker, correlations were found in the case of IgG. Of note, the occurrence of IgA antibodies was also significantly more frequent than that of IgG antibodies. Interestingly, elevated IgA levels were associated with disease severity based on traditional measures (Child–Pugh stages), which rather correspond to chronic progression. This observation is in line with the slower response time of the adaptive immune system. On the other hand, the decreased level of IgG correlated with severity according to CLIF‐C AD score and the presence of ACLF, which parameters were designed specifically for AD patients [18, 20]. Accordingly, increased IgA levels are likely a result of chronic stimulation at mucosal surfaces, whereas decreased IgG levels may be a result of an imbalance between acute consumption and insufficient production due to cirrhosis‐associated immune dysfunction.

Furthermore, we previously demonstrated that certain disease‐related target‐specific IgA (but not IgG) autoantibodies contributed to poorer long‐term outcomes in stable cirrhotic outpatients [7, 8]. However, these effects have never been investigated in the context of AD and its most severe form: ACLF syndrome. In the present study, an increased ratio of total‐IgA to total‐IgG levels was found to be associated with 28‐day mortality. This indicates a more pronounced shift in the antibody production from IgG to IgA in nonsurvivors, supporting BT‐triggered immune dysregulation in these patients. However, none of the measured markers were associated with 90‐day mortality in the whole patient population. On the other hand, in ACLF patients, increased sIgA levels were strongly associated with an increased 90‐day mortality rate in both cohorts investigated. Paradoxically, generally decreased sIgA levels were demonstrated in the ACLF population compared to both cross‐sectionally to non‐ACLF patients with CLIF‐C AD scores ≥ 50 and longitudinally to their own pre‐ACLF state. This observation aligns with previous reports of reduced sIgA levels in acute liver failure [5, 6]. Additionally, our data revealed an association between renal failure and decreased sIgA levels, which was more pronounced in the discovery cohort (data not shown), where patients typically presented with fewer concurrent organ failures than those in the validation cohort. This finding is consistent with literature demonstrating that both mRNA and protein expression of the polymeric‐Ig receptor—essential for transepithelial IgA transport—are markedly reduced in kidney tissue during ischaemic kidney injury, resulting in decreased urinary sIgA levels [35]. While the precise mechanistic explanation for decreased circulating sIgA requires further investigation, this finding demonstrates that impaired epithelial transport capacity may contribute to reduced sIgA levels. On the other hand, there are two possible—and not exclusive—explanations. First, a certain degree of correlation can be assumed between renal and intestinal insults as the haemodynamic disturbances associated with AD cirrhosis may simultaneously induce renal ischaemia—leading to AKI [36]—while causing circulatory congestion, oedema and consequent hypoxia in the intestines that disrupts mucosal integrity [37, 38]. The second is that kidney failure alone represents the least severe form of ACLF (grade 1) that might only be associated with functional impairment (i.e., downregulated transportation) in the intestines.

Thus, the key to reconciling the apparent contradiction between generally decreased—compared to non‐ACLF patients with CLIF‐C AD scores ≥ 50—and mortality‐associated increased sIgA levels in ACLF lies in understanding the three‐step process leading to sIgA appearance in circulation: (1) mucosal IgA production, (2) active secretion into the intestinal lumen via the polymeric‐Ig receptor (i.e., binding with the secretory component) and (3) subsequent translocation from the lumen back into circulation. Based on this framework, we propose the following hypothesis (Figure S1). Initially, dysbiosis and bacterial overgrowth associated with increasing disease severity—reflected by Child–Pugh stages, MELD and CLIF‐C AD scores—trigger IgA production and sIgA secretion. However, in ACLF, gut mucosal dysfunction develops, leading to decreased sIgA assembly. Since ACLF was not associated with decreased total IgA levels, epithelial transport mechanisms are likely disrupted—as observed in ischaemic kidney injury—rather than antibody production itself by plasma cells. With further disease progression—reflected by increasing CLIF‐C ACLF scores—serum sIgA levels increase again. This elevation cannot reasonably be attributed to enhanced IgA production by an exhausted immune system or increased sIgA reuptake by damaged epithelial cells. Instead, it most likely results from severe gut barrier injury leading to accelerated passive leakage of sIgA from the intestinal lumen into circulation. Since sIgA would not be the only molecule entering circulation this way (i.e., BT), the loss of barrier function (i.e., gut barrier failure) results in a severe negative impact on survival. Therefore, we propose that increased sIgA levels in ACLF patients indicate a severely injured and permeable gut barrier, in other words, failure of the gut barrier. Therefore, high sIgA concentration could define gut barrier failure in patients with already established ACLF and increase the patients' ACLF grade (→two or multiple organ failures). In our united ACLF cohort, based on sIgA levels, nine patients could have been recategorised from ACLF 1 to 2, of whom five patients (55.6%) died. In contrast, 22 patients could have been recategorised from ACLF 2 to 3, of whom 19 patients (86.4%) died (data not shown). This is most probably due to the multimodal dynamics of serum sIgA change (increases parallel with AD, decreases in ACLF, but increases again according to ACLF grade and mortality). Therefore, sIgA levels need to be interpreted together with severity measures, especially in the case of ACLF 1, but may provide valuable information to further refine patients' risk stratification by defining gut barrier failure in ACLF.

Our study describes previously unrecognised alterations in antibody‐mediated immune responses in acutely decompensated cirrhosis and identifies a promising prognostic biomarker in ACLF, which are strengths of this article. While our findings are primarily descriptive, we integrated them with existing literature to develop a hypothetical framework that connects previously disparate observations and advances our understanding of pathological processes. The relatively small sample size of the two ACLF cohorts and the reliance on an internal validation cohort are limitations of this study. Nonetheless, the association between sIgA and 90‐day mortality was robust in both ACLF populations. This supports the potential generalisability of our findings, albeit further validation in larger, external cohorts is needed.

## 5 Conclusion

Enhanced BT‐triggered immune activation is indicated by increased total‐IgA levels in association with the occurrence of disease‐related target‐specific IgA antibodies. However, these markers were not predictive of 90‐day outcomes in AD, in contrast to their long‐term predictive capability in outpatients. This might be due to a slower reaction time of the adaptive immunity. On the other hand, due to the cross‐barrier transport of sIgA, it is able to indicate permeability and failure of the gut barrier. Therefore, increased serum sIgA level is a promising marker to enhance risk stratification and predict 90‐day mortality in ACLF patients.

## Author Contributions

M.P. and D.T. conceptualised the study. D.T., B.B. and A.C. performed literature search. B.B. collected clinical data of the patients. B.B., I.T., Z.V., N.S., P.K. and T.D. treated the patients in the discovery cohort and collected their samples. A.C. and P.A.S. performed and oversaw laboratory measurements. D.T. analysed the results, compared them to the literature and wrote the article with B.B. and M.P. W.L., M.J.C. and J.T. provided expert opinion. All authors reviewed and approved the final manuscript.

## Conflicts of Interest

The authors declare no conflicts of interest. This work reflects only the author's view, and the European Commission is not responsible for any use that may be made of the information it contains.

## Supporting information


**Figure S1:** Summary of the hypothesis on the pathophysiology of sIgA level change in patients with cirrhosis and acute‐on‐chronic liver failure. In cirrhosis, enhanced bacterial burden triggers the production of IgA and secretion of sIgA. However, in ACLF, due to the impairment of gut mucosal functions particularly the transport of (s)IgA, the serum level of sIgA decreases. When the integrity of the barrier is also damaged sIgA leaks back to the circulation along with bacteria and bacterial products that increases mortality risk.
**Table S1:**. Correlation analysis between total immunoglobulin levels, routine laboratory parameters, macrophage markers, clinical scores and levels of individual disease‐related target‐specific antibodies.
**Table S2:** Correlation analysis between free light chains, sIgA and Endocab IgA levels, routine laboratory parameters, macrophage markers, clinical scores and levels of total immunoglobulins and individual disease‐related target‐specific antibodies.
**Table S3:** Correlation analysis between ASCA and anti‐F‐actin antibodies, routine laboratory parameters, macrophage markers, clinical scores and other antibody markers.
**Table S4:** Correlation analysis between Anti‐Gliadin and Anti‐GP2 antibodies, routine laboratory parameters, macrophage markers, clinical scores and other antibody markers.
**Table S5:** Association between alcoholic aetiology and levels of measured antibodies.
**Table S6:** Association between alcoholic aetiology and frequency of disease‐related target‐specific antibodies.
**Table S7:** Correlation between number (#) of disease‐related target‐specific antibodies (ABs) and other immunoglobulin (Ig) markers.
**Table S8:** Immunoglobulin A antibody levels are correlated with Child–Pugh score.
**Table S9:** Clinical and laboratory characteristics of patients with acute‐on‐chronic liver failure (ACLF).
**Table S10:** High serum sIgA levels according to both discovery and validation cohort cut‐offs predicts 90‐day mortality independently of ACLF grade in the merged ACLF cohort (*n* = 87).
**Table S11:** Liver, cerebral, circulatory and respiratory failures are associated with increased, while renal failure is associated with decreased serum secretory (s)IgA levels in patients with acute‐on‐chronic liver failure.
**Table S12:** No statistically significant difference was found in 90‐day mortality rates between acute‐on‐chronic liver failure (ACLF) patients receiving and not receiving nonselective beta blockers (NSBBs).
**Table S13:** Secretory immunoglobulin A (sIgA) serum levels demonstrated excellent discriminatory capability for 90‐day mortality in patients not receiving nonselective beta blockers (NSBBs) but could not differentiate between patients on this treatment.

**Data S1:** Supporting Information S1.

## Data Availability

The data that support the findings of this study are available from the corresponding author upon reasonable request.
