# Supplementary material for: Secretory IgA Is a Key Marker Among Gut Barrier Dysfunction‐Related Immunoglobulins Predicting Outcomes in ACLF
Source: Liver Int. 2025 Sep 13;45(10):e70350. doi: 10.1111/liv.70350 (PMC12432679; doi:10.1111/liv.70350)
Supplement: Supplementary file 1 — Figure S1: Summary of the hypothesis on the pathophysiology of sIgA level change in patients with cirrhosis and acute‐on‐chronic liver failure. In cirrhosis, enhanced bacterial burden triggers the production of IgA and secretion of sIgA. However, in ACLF, due to the impairment of gut mucosal functions particularly the transport of (s)IgA, the serum level of sIgA decreases. When the integrity of the barrier is also damaged sIgA leaks back to the circulation along with bacteria and bacterial products that increases mortality risk. Table S1:. Correlation analysis between total immunoglobulin levels, routine laboratory parameters, macrophage markers, clinical scores and levels of individual disease‐related target‐specific antibodies. Table S2: Correlation analysis between free light chains, sIgA and Endocab IgA levels, routine laboratory parameters, macrophage markers, clinical scores and levels of total immunoglobulins and individual disease‐related target‐specific antibodies. Table S3: Correlation analysis between ASCA and anti‐F‐actin antibodies, routine laboratory parameters, macrophage markers, clinical scores and other antibody markers. Table S4: Correlation analysis between Anti‐Gliadin and Anti‐GP2 antibodies, routine laboratory parameters, macrophage markers, clinical scores and other antibody markers. Table S5: Association between alcoholic aetiology and levels of measured antibodies. Table S6: Association between alcoholic aetiology and frequency of disease‐related target‐specific antibodies. Table S7: Correlation between number (#) of disease‐related target‐specific antibodies (ABs) and other immunoglobulin (Ig) markers. Table S8: Immunoglobulin A antibody levels are correlated with Child–Pugh score. Table S9: Clinical and laboratory characteristics of patients with acute‐on‐chronic liver failure (ACLF). Table S10: High serum sIgA levels according to both discovery and validation cohort cut‐offs predicts 90‐day mortality independently of ACLF grade in the me [file LIV-45-0-s001.docx]

**Supplementary materials**

**Secretory IgA is a key marker among gut barrier dysfunction-related immunoglobulins predicting outcomes in acute-on-chronic liver failure**

Boglarka Balogh^φ^, David Tornai^φ^, Aniko Csillag, Istvan Tornai, Zsuzsana Vitalis, Patricia Kovats, Peter Antal-Szalmas, Tamas Dinya, Wim Laleman, Minneke J Coenraad, Jonel Trebicka, Maria Papp on behalf of MICROB-PREDICT and PREDICT study group of the EASL-CLIF consortium

^φ^Boglarka Balogh and David Tornai contributed equally to the work, and both should be considered as first authors.

**Table of contents**

Supplementary Figure 1…………………………………………………..2

Supplementary Table 1. ………………………………………………….3

Supplementary Table 2. ………………………………………………….4

Supplementary Table 3. ………………………………………………….5

Supplementary Table 4. ………………………………………………….6

Supplementary Table 5. ………………………………………………….7

Supplementary Table 6. ………………………………………………….8

Supplementary Table 7. ………………………………………………….8

Supplementary Table 8. ………………………………………………….9

Supplementary Table 9. …………………………………………………10

Supplementary Table 10. ………………………………………………..10

Supplementary Table 11. ………………………………………………..10

Supplementary Table 12. ………………………………………………..11

Supplementary Table 13. ………………………………………………..11


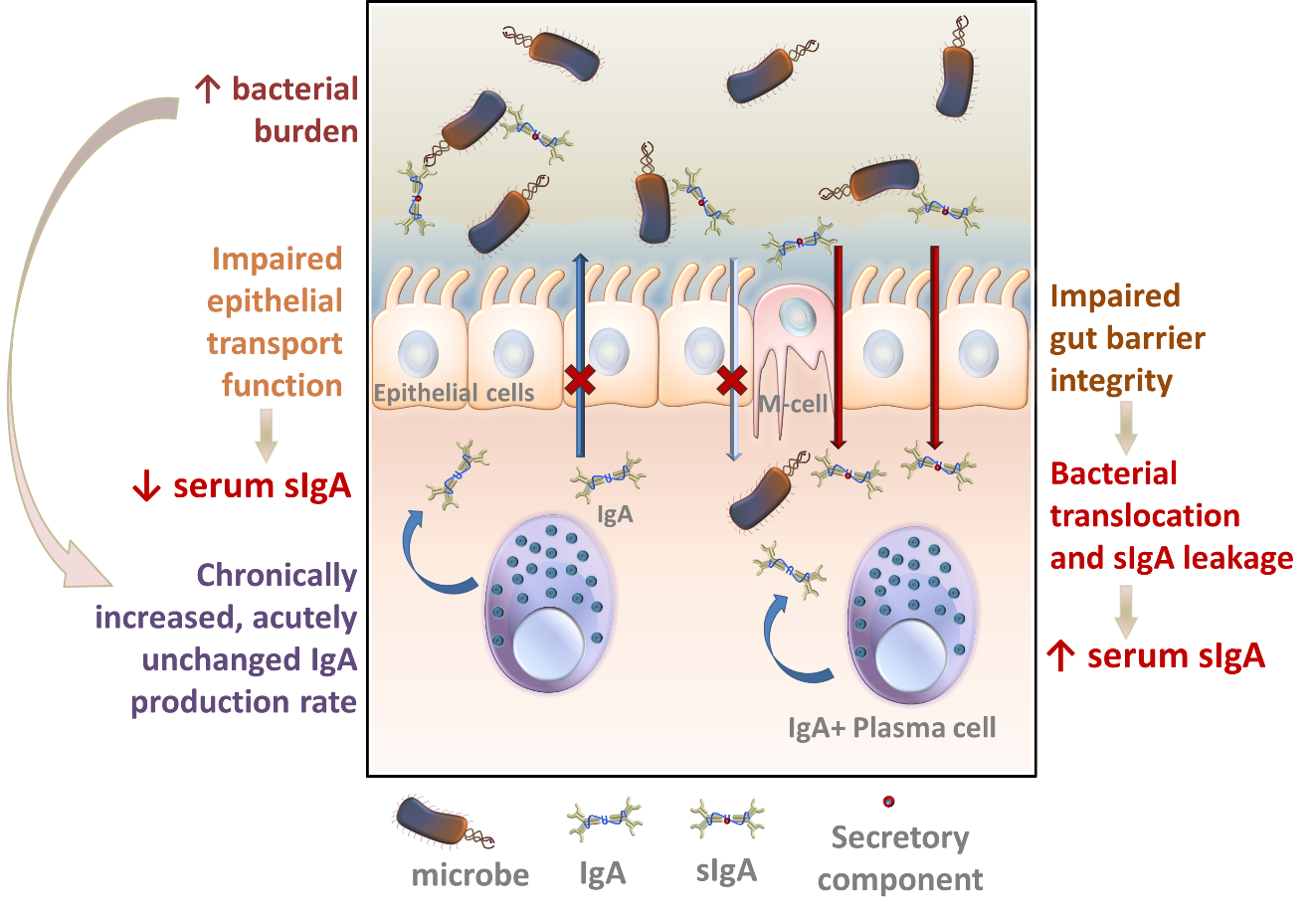


**Supplementary Figure 1. Summary of the hypothesis on the pathophysiology of sIgA level change in patients with cirrhosis and acute-on-chronic liver failure.** In cirrhosis, enhanced bacterial burden triggers the production of IgA and secretion of sIgA. However, in ACLF, due to the impairment of gut mucosal functions particularly the transport of (s)IgA, the serum level of sIgA decreases. When the integrity of the barrier is also damaged sIgA leaks back to the circulation along with bacteria and bacterial products that increases mortality risk.

**Supplementary Table 1. Correlation analysis between t*otal immunoglobulin levels, routine laboratory parameters, macrophage markers, clinical scores and levels of individual disease-related target-specific antibodies***

|  | Total IgA | | Total IgG | | Total IgM | |
| --- | --- | --- | --- | --- | --- | --- |
|  | r | p | r | p | r | p |
| Bilirubin | 0.127 | 0.152 | 0.029 | 0.747 | 0.319 | **<0.001** |
| Albumin | -0.364 | **<0.001** | -0.148 | 0.096 | -0.002 | 0.980 |
| INR | 0.324 | **<0.001** | 0.094 | 0.289 | 0.224 | **0.011** |
| Creatinine | -0.097 | 0.277 | -0.100 | 0.264 | -0.258 | **0.003** |
| AST | 0.248 | **0.005** | 0.085 | 0.343 | 0.356 | **<0.001** |
| ALT | 0.000 | 0.999 | -0.073 | 0.418 | 0.258 | **0.004** |
| ALP | 0.080 | 0.372 | 0.012 | 0.896 | 0.173 | 0.052 |
| γGT | 0.010 | 0.918 | -0.153 | 0.101 | 0.129 | 0.164 |
| WBC | -0.254 | **0.004** | -0.293 | **<0.001** | 0.047 | 0.601 |
| Neutrophils | -0.253 | **0.004** | -0.275 | **0.002** | 0.067 | 0.450 |
| Platelets | -0.268 | **0.002** | -0.187 | **0.034** | -0.139 | 0.117 |
| CRP | -0.116 | 0.191 | -0.154 | 0.082 | -0.007 | 0.939 |
| PCT | -0.156 | 0.083 | -0.265 | **0.003** | 0.089 | 0.323 |
| Presepsin | -0.106 | 0.240 | -0.176 | **0.049** | -0.130 | 0.147 |
| sCD163 | 0.199 | **0.028** | 0.106 | 0.248 | 0.294 | **0.001** |
| sCD206 | 0.387 | **<0.001** | 0.262 | **0.004** | 0.179 | 0.050 |
| MELD | 0.153 | 0.085 | -0.049 | 0.584 | 0.156 | 0.078 |
| Child-Pugh | 0.340 | **<0.001** | 0.084 | 0.344 | 0.137 | 0.123 |
| CLIF-C AD-score | 0.006 | 0.948 | -0.146 | 0.131 | -0.009 | 0.931 |
| CLIF-C ACLF-score | 0.173 | 0.465 | 0.028 | 0.907 | 0.161 | 0.497 |
| Total IgA |  |  | 0.536 | **<0.001** | 0.215 | **0.015** |
| Total IgG | 0.536 | **<0.001** |  |  | 0.314 | **<0.001** |
| Total IgM | 0.215 | **0.015** | 0.314 | **<0.001** |  |  |
| Kappa Ig LC | 0.401 | **<0.001** | 0.435 | **<0.001** | -0.019 | 0.836 |
| Lambda Ig LC | 0.559 | **<0.001** | 0.570 | **<0.001** | 0.008 | 0.933 |
| sIgA | 0.175 | **0.049** | 0.113 | 0.206 | 0.151 | 0.089 |
| EndoCab IgA | 0.706 | **<0.001** | 0.481 | **<0.001** | 0.271 | **0.002** |
| ASCA IgA | 0.580 | **<0.001** | 0.207 | **0.019** | 0.089 | 0.318 |
| ASCA IgG | 0.102 | 0.253 | 0.193 | **0.029** | 0.096 | 0.283 |
| Anti-F-actin IgA | 0.672 | **<0.001** | 0.426 | **<0.001** | 0.276 | **0.002** |
| Anti-F-actin IgG | 0.234 | **0.008** | 0.399 | **<0.001** | 0.308 | **<0.001** |
| Anti-Gliadin IgA | 0.401 | **<0.001** | 0.289 | **<0.001** | 0.258 | **0.003** |
| Anti-Gliadin IgG | 0.197 | **0.026** | 0.160 | 0.072 | 0.026 | 0.775 |
| Anti-GP2 IgA | 0.335 | **<0.001** | 0.094 | 0.291 | 0.024 | 0.785 |
| Anti-GP2 IgG | 0.100 | 0.260 | -0.012 | 0.892 | -0.015 | 0.870 |

Total immunoglobulin levels were correlated with the levels of individual disease-related target-specific antibodies. CLIF-C: Chronic-Liver Failure Consortium; ACLF: acute-on-chronic liver failure; AD: acute decompensation; MELD: Model for End-Stage Liver Disease, INR: international normalized ratio; AST: aspartate aminotransferase, ALT: alanine aminotransferase, ALP: alkaline phosphatase, γGT: gamma-glutamyl transferase, WBC: white blood cell count; CRP: C-reactive protein; PCT: procalcitonin; Ig: immunoglobulin; LC: light chain; sIgA: secretory IgA, EndoCab: endotoxin core antibody; ASCA: anti-*Saccharomyces* cerevisiae antibody; GP2: pancreatic glycoprotein 2.

**Supplementary Table 2. Correlation analysis between free light chains, sIgA and Endocab IgA levels*, routine laboratory parameters, macrophage markers, clinical scores and levels of total immunoglobulins and individual disease-related target-specific antibodies***

|  | Kappa Ig LC | | Lambda Ig LC | | sIgA | | EndoCab IgA | |
| --- | --- | --- | --- | --- | --- | --- | --- | --- |
|  | r | p | r | p | r | p | r | p |
| Bilirubin | -0.228 | **0.010** | -0.074 | 0.404 | 0.572 | **<0.001** | 0.147 | 0.098 |
| Albumin | -0.250 | **0.004** | -0.373 | **<0.001** | -0.207 | **0.019** | -0.360 | **<0.001** |
| INR | 0.010 | 0.913 | 0.132 | 0.138 | 0.398 | **<0.001** | 0.268 | **0.002** |
| Creatinine | 0.435 | **<0.001** | 0.237 | **0.007** | -0.195 | **0.027** | -0.126 | 0.158 |
| AST | -0.217 | **0.015** | -0.075 | 0.407 | 0.725 | **<0.001** | 0.193 | **0.030** |
| ALT | -0.284 | **0.001** | -0.253 | **0.004** | 0.576 | **<0.001** | 0.047 | 0.603 |
| ALP | -0.118 | 0.189 | -0.040 | 0.659 | 0.656 | **<0.001** | 0.133 | 0.137 |
| γGT | -0.290 | **0.002** | -0.237 | **0.010** | 0.713 | **<0.001** | 0.035 | 0.706 |
| WBC | -0.177 | **0.046** | -0.177 | **0.045** | 0.155 | 0.081 | -0.229 | **0.010** |
| Neutrophils | -0.193 | **0.030** | -0.196 | **0.026** | 0.156 | 0.078 | -0.235 | **0.008** |
| Platelets | -0.073 | 0.412 | -0.043 | 0.631 | -0.053 | 0.552 | -0.239 | **0.007** |
| CRP | 0.039 | 0.662 | -0.047 | 0.600 | 0.276 | **0.002** | 0.016 | 0.861 |
| PCT | 0.023 | 0.803 | -0.077 | 0.393 | 0.383 | **<0.001** | -0.162 | 0.072 |
| Presepsin | 0.167 | 0.063 | 0.098 | 0.278 | 0.363 | **<0.001** | -0.048 | 0.594 |
| sCD163 | 0.024 | 0.796 | 0.111 | 0.225 | 0.420 | **<0.001** | 0.136 | 0.138 |
| sCD206 | 0.208 | **0.022** | 0.261 | **0.004** | 0.179 | **0.049** | 0.435 | **<0.001** |
| MELD | -0.001 | 0.993 | 0.099 | 0.265 | 0.403 | **<0.001** | 0.139 | 0.119 |
| Child-Pugh | 0.042 | 0.638 | 0.162 | 0.068 | 0.361 | **<0.001** | 0.333 | **<0.001** |
| CLIF-C AD-score | 0.094 | 0.333 | 0.076 | 0.436 | 0.181 | 0.061 | -0.067 | 0.491 |
| CLIF-C ACLF-sc. | -0.144 | 0.545 | -0.090 | 0.705 | 0.620 | **0.004** | -0.017 | 0.942 |
| Total IgA | 0.401 | **<0.001** | 0.559 | **<0.001** | 0.175 | **0.049** | 0.706 | **<0.001** |
| Total IgG | 0.435 | **<0.001** | 0.570 | **<0.001** | 0.113 | 0.206 | 0.481 | **<0.001** |
| Total IgM | -0.019 | 0.836 | 0.008 | 0.933 | 0.151 | 0.089 | 0.271 | **0.002** |
| Kappa Ig LC |  |  | 0.715 | **<0.001** | -0.083 | 0.352 | 0.290 | **<0.001** |
| Lambda Ig LC | 0.715 | **<0.001** |  |  | -0.028 | 0.754 | 0.426 | **<0.001** |
| sIgA | -0.083 | 0.352 | -0.028 | 0.754 |  |  | 0.226 | **0.010** |
| EndoCab IgA | 0.290 | **<0.001** | 0.426 | **<0.001** | 0.226 | **0.010** |  |  |
| ASCA IgA | 0.268 | **0.002** | 0.267 | **0.002** | 0.167 | 0.060 | 0.419 | **<0.001** |
| ASCA IgG | 0.035 | 0.692 | 0.024 | 0.787 | -0.023 | 0.795 | 0.159 | 0.073 |
| Anti-F-actin IgA | 0.432 | **<0.001** | 0.521 | **<0.001** | 0.221 | **0.013** | 0.619 | **<0.001** |
| Anti-F-actin IgG | 0.223 | **0.012** | 0.178 | **0.045** | 0.210 | **0.018** | 0.245 | **0.006** |
| Anti-Gliadin IgA | 0.074 | 0.409 | 0.197 | **0.027** | 0.010 | 0.909 | 0.342 | **<0.001** |
| Anti-Gliadin IgG | -0.034 | 0.705 | -0.019 | 0.836 | -0.021 | 0.819 | 0.128 | 0.152 |
| Anti-GP2 IgA | 0.141 | 0.113 | 0.200 | **0.024** | 0.093 | 0.299 | 0.302 | **<0.001** |
| Anti-GP2 IgG | 0.135 | 0.129 | 0.159 | 0.072 | -0.024 | 0.786 | 0.235 | **0.008** |

CLIF-C: Chronic-Liver Failure Consortium; ACLF: acute-on-chronic liver failure; AD: acute decompensation; MELD: Model for End-Stage Liver Disease, INR: international normalized ratio; AST: aspartate aminotransferase, ALT: alanine aminotransferase, ALP: alkaline phosphatase, γGT: gamma-glutamyl transferase, WBC: white blood cell count; CRP: C-reactive protein; PCT: procalcitonin; Ig: immunoglobulin; LC: light chain; sIgA: secretory IgA, EndoCab: endotoxin core antibody; ASCA: anti-*Saccharomyces* cerevisiae antibody; GP2: pancreatic glycoprotein 2.

**Supplementary Table 3. Correlation analysis between ASCA and anti-F-actin antibodies*, routine laboratory parameters, macrophage markers, clinical scores and other antibody markers***

|  | ASCA IgA | | ASCA IgG | | Anti-F-actin IgA | | Anti-F-actin IgG | |  |
| --- | --- | --- | --- | --- | --- | --- | --- | --- | --- |
|  | r | p | r | p | r | p | r | p | |
| Bilirubin | 0.164 | 0.065 | -0.065 | 0.467 | 0.195 | **0.028** | 0.148 | 0.098 | |
| Albumin | -0.304 | **<0.001** | -0.222 | **0.012** | -0.427 | **<0.001** | -0.138 | 0.122 | |
| INR | 0.224 | **0.011** | 0.036 | 0.686 | 0.348 | **<0.001** | 0.123 | 0.169 | |
| Creatinine | -0.115 | 0.196 | -0.088 | 0.323 | -0.100 | 0.265 | -0.117 | 0.189 | |
| AST | 0.186 | **0.038** | -0.100 | 0.268 | 0.264 | **0.003** | 0.168 | 0.061 | |
| ALT | 0.107 | 0.230 | -0.001 | 0.988 | 0.042 | 0.642 | 0.156 | 0.081 | |
| ALP | 0.104 | 0.249 | 0.009 | 0.918 | 0.124 | 0.169 | 0.118 | 0.189 | |
| γGT | 0.050 | 0.592 | -0.121 | 0.192 | 0.059 | 0.528 | 0.085 | 0.363 | |
| WBC | -0.128 | 0.150 | -0.074 | 0.406 | -0.093 | 0.299 | -0.110 | 0.218 | |
| Neutrophils | -0.161 | 0.069 | -0.075 | 0.399 | -0.087 | 0.332 | -0.124 | 0.166 | |
| Platelets | -0.267 | **0.002** | -0.020 | 0.820 | -0.282 | **0.001** | -0.084 | 0.347 | |
| CRP | 0.009 | 0.922 | -0.009 | 0.920 | 0.003 | 0.970 | 0.053 | 0.552 | |
| PCT | -0.026 | 0.776 | -0.083 | 0.360 | 0.007 | 0.935 | 0.094 | 0.301 | |
| Presepsin | -0.073 | 0.418 | -0.121 | 0.178 | 0.047 | 0.602 | -0.028 | 0.759 | |
| sCD163 | 0.102 | 0.268 | -0.023 | 0.800 | 0.246 | **0.007** | 0.172 | 0.060 | |
| sCD206 | 0.259 | **0.004** | 0.074 | 0.422 | 0.369 | **<0.001** | 0.170 | 0.063 | |
| MELD | 0.096 | 0.282 | -0.077 | 0.386 | 0.210 | **0.018** | 0.054 | 0.544 | |
| Child-Pugh | 0.265 | **0.003** | 0.103 | 0.247 | 0.375 | **<0.001** | 0.152 | 0.089 | |
| CLIF-C AD-score | -0.054 | 0.579 | -0.151 | 0.119 | 0.065 | 0.503 | 0.039 | 0.693 | |
| CLIF-C ACLF-sc. | 0.009 | 0.970 | -0.132 | 0.581 | 0.224 | 0.342 | -0.144 | 0.544 | |
| Total IgA | 0.580 | **<0.001** | 0.102 | 0.253 | 0.672 | **<0.001** | 0.234 | **0.008** | |
| Total IgG | 0.207 | **0.019** | 0.193 | **0.029** | 0.426 | **<0.001** | 0.399 | **<0.001** | |
| Total IgM | 0.089 | 0.318 | 0.096 | 0.283 | 0.276 | **0.002** | 0.308 | **<0.001** | |
| Kappa Ig LC | 0.268 | **0.002** | 0.035 | 0.692 | 0.432 | **<0.001** | 0.223 | **0.012** | |
| Lambda Ig LC | 0.267 | **0.002** | 0.024 | 0.787 | 0.521 | **<0.001** | 0.178 | **0.045** | |
| sIgA | 0.167 | 0.060 | -0.023 | 0.795 | 0.221 | **0.013** | 0.210 | **0.018** | |
| EndoCab IgA | 0.419 | **<0.001** | 0.159 | 0.073 | 0.619 | **<0.001** | 0.245 | **0.006** | |
| ASCA IgA |  |  | 0.256 | **0.004** | 0.549 | **<0.001** | 0.188 | **0.034** | |
| ASCA IgG | 0.256 | **0.004** |  |  | 0.098 | 0.271 | 0.113 | 0.205 | |
| Anti-F-actin IgA | 0.549 | **<0.001** | 0.098 | 0.271 |  |  | 0.404 | **<0.001** | |
| Anti-F-actin IgG | 0.188 | **0.034** | 0.113 | 0.205 | 0.404 | **<0.001** |  |  | |
| Anti-Gliadin IgA | 0.293 | **<0.001** | 0.008 | 0.934 | 0.402 | **<0.001** | 0.165 | 0.065 | |
| Anti-Gliadin IgG | 0.133 | 0.135 | 0.102 | 0.254 | 0.061 | 0.498 | -0.010 | 0.909 | |
| Anti-GP2 IgA | 0.322 | **<0.001** | -0.022 | 0.806 | 0.378 | **<0.001** | 0.120 | 0.179 | |
| Anti-GP2 IgG | 0.054 | 0.548 | 0.038 | 0.674 | 0.136 | 0.128 | 0.069 | 0.441 | |

CLIF-C: Chronic-Liver Failure Consortium; ACLF: acute-on-chronic liver failure; AD: acute decompensation; MELD: Model for End-Stage Liver Disease, INR: international normalized ratio; AST: aspartate aminotransferase, ALT: alanine aminotransferase, ALP: alkaline phosphatase, γGT: gamma-glutamyl transferase, WBC: white blood cell count; CRP: C-reactive protein; PCT: procalcitonin; Ig: immunoglobulin; LC: light chain; sIgA: secretory IgA, EndoCab: endotoxin core antibody; ASCA: anti-*Saccharomyces* cerevisiae antibody; GP2: pancreatic glycoprotein 2.

**Supplementary Table 4. Correlation analysis between Anti-Gliadin and Anti-GP2 antibodies*, routine laboratory parameters, macrophage markers, clinical scores and other antibody markers***

|  | Anti-Gliadin IgA | | Anti-Gliadin IgG | | Anti-GP2 IgA | | Anti-GP2 IgG | |
| --- | --- | --- | --- | --- | --- | --- | --- | --- |
|  | r | p | r | p | r | p | r | p |
| Bilirubin | 0.172 | 0.054 | -0.024 | 0.790 | 0.067 | 0.451 | -0.129 | 0.147 |
| Albumin | -0.107 | 0.234 | 0.019 | 0.832 | -0.159 | 0.073 | -0.116 | 0.191 |
| INR | 0.194 | **0.029** | -0.005 | 0.955 | 0.177 | **0.046** | -0.023 | 0.795 |
| Creatinine | -0.162 | 0.070 | -0.072 | 0.424 | -0.055 | 0.536 | 0.160 | 0.072 |
| AST | 0.087 | 0.332 | -0.016 | 0.861 | 0.031 | 0.735 | -0.047 | 0.600 |
| ALT | 0.033 | 0.712 | -0.025 | 0.781 | -0.030 | 0.737 | -0.020 | 0.820 |
| ALP | 0.020 | 0.825 | 0.099 | 0.270 | 0.008 | 0.933 | 0.113 | 0.209 |
| γGT | -0.052 | 0.579 | -0.062 | 0.512 | -0.093 | 0.319 | 0.010 | 0.915 |
| WBC | -0.093 | 0.301 | -0.309 | **<0.001** | -0.223 | **0.012** | -0.243 | **0.006** |
| Neutrophils | -0.130 | 0.147 | -0.270 | **0.002** | -0.192 | **0.030** | -0.251 | **0.004** |
| Platelets | -0.319 | **<0.001** | -0.298 | **<0.001** | -0.184 | **0.038** | -0.097 | 0.274 |
| CRP | 0.079 | 0.378 | -0.148 | 0.096 | -0.079 | 0.378 | -0.008 | 0.928 |
| PCT | -0.116 | 0.203 | -0.082 | 0.370 | -0.093 | 0.304 | -0.011 | 0.905 |
| Presepsin | -0.139 | 0.122 | -0.170 | 0.059 | -0.031 | 0.732 | 0.102 | 0.256 |
| sCD163 | 0.121 | 0.189 | -0.008 | 0.932 | 0.075 | 0.413 | -0.056 | 0.544 |
| sCD206 | 0.300 | **<0.001** | 0.227 | **0.013** | 0.370 | **<0.001** | 0.070 | 0.444 |
| MELD | 0.128 | 0.151 | -0.042 | 0.642 | 0.076 | 0.392 | 0.029 | 0.748 |
| Child-Pugh | 0.171 | 0.055 | 0.032 | 0.723 | 0.218 | **0.013** | 0.039 | 0.661 |
| CLIF-C AD-score | -0.033 | 0.737 | -0.185 | 0.057 | -0.053 | 0.586 | -0.138 | 0.154 |
| CLIF-C ACLF-sc. | 0.244 | 0.299 | 0.263 | 0.263 | -0.184 | 0.437 | -0.403 | 0.079 |
| Total IgA | 0.401 | **<0.001** | 0.197 | **0.026** | 0.335 | **<0.001** | 0.100 | 0.260 |
| Total IgG | 0.289 | **<0.001** | 0.160 | 0.072 | 0.094 | 0.291 | -0.012 | 0.892 |
| Total IgM | 0.258 | **0.003** | 0.026 | 0.775 | 0.024 | 0.785 | -0.015 | 0.870 |
| Kappa Ig LC | 0.074 | 0.409 | -0.034 | 0.705 | 0.141 | 0.113 | 0.135 | 0.129 |
| Lambda Ig LC | 0.197 | **0.027** | -0.019 | 0.836 | 0.200 | **0.024** | 0.159 | 0.072 |
| sIgA | 0.010 | 0.909 | -0.021 | 0.819 | 0.093 | 0.299 | -0.024 | 0.786 |
| EndoCab IgA | 0.342 | **<0.001** | 0.128 | 0.152 | 0.302 | **<0.001** | 0.235 | **0.008** |
| ASCA IgA | 0.293 | **<0.001** | 0.133 | 0.135 | 0.322 | **<0.001** | 0.054 | 0.548 |
| ASCA IgG | 0.008 | 0.934 | 0.102 | 0.254 | -0.022 | 0.806 | 0.038 | 0.674 |
| Anti-F-actin IgA | 0.402 | **<0.001** | 0.061 | 0.498 | 0.378 | **<0.001** | 0.136 | 0.128 |
| Anti-F-actin IgG | 0.165 | 0.065 | -0.010 | 0.909 | 0.120 | 0.179 | 0.069 | 0.441 |
| Anti-Gliadin IgA |  |  | 0.391 | **<0.001** | 0.293 | **<0.001** | 0.030 | 0.740 |
| Anti-Gliadin IgG | 0.391 | **<0.001** |  |  | 0.201 | **0.024** | 0.026 | 0.775 |
| Anti-GP2 IgA | 0.293 | **<0.001** | 0.201 | **0.024** |  |  | 0.262 | **0.003** |
| Anti-GP2 IgG | 0.030 | 0.740 | 0.026 | 0.775 | 0.262 | **0.003** |  |  |

CLIF-C: Chronic-Liver Failure Consortium; ACLF: acute-on-chronic liver failure; AD: acute decompensation; MELD: Model for End-Stage Liver Disease, INR: international normalized ratio; AST: aspartate aminotransferase, ALT: alanine aminotransferase, ALP: alkaline phosphatase, γGT: gamma-glutamyl transferase, WBC: white blood cell count; CRP: C-reactive protein; PCT: procalcitonin; Ig: immunoglobulin; LC: light chain; sIgA: secretory IgA, EndoCab: endotoxin core antibody; ASCA: anti-*Saccharomyces* cerevisiae antibody; GP2: pancreatic glycoprotein 2.

**Supplementary Table 5. Association between alcoholic etiology and levels of measured antibodies**

|  | Non-alcoholic | Alcoholic | p |
| --- | --- | --- | --- |
| Total IgA | 3.28 (1.68-6.23) | 5.75 (4.02-9.56) | **<0.001** |
| Total IgG | 18.1 (12.13-20.5) | 16.4 (11.4-20.55) | 0.353 |
| Total IgM | 1.54 (0.98-2.74) | 1.48 (1-2.37) | 0.727 |
| Kappa Ig LC | 22.3 (7.84-38.63) | 22.15 (14.9-33.05) | 0.655 |
| Lambda Ig LC | 29.1 (19.45-44.18) | 34.95 (23.68-49.4) | 0.303 |
| sIgA | 15.6 (9.81-27.31) | 33.08 (14.7-63.28) | **0.009** |
| EndoCab IgA | 118.23 (66.77-274) | 177.46 (116.13-321.05) | 0.053 |
| ASCA IgA | 0.00 (0.00-0.00) | 1.16 (0-11.91) | **0.010** |
| ASCA IgG | 0.00 (0.00-0.46) | 0.00 (0.00-0.00) | 0.419 |
| Anti-F-actin IgA | 0.00 (0.00-21.12) | 10.34 (0-28.91) | 0.096 |
| Anti-F-actin IgG | 0.00 (0.00-3.21) | 0.00 (0.00-3.77) | 0.701 |
| Anti-Gliadin IgA | 0.00 (0.00-146.82) | 2.17 (0-28.21) | 0.655 |
| Anti-Gliadin IgG | 0.00 (0.00-0.00) | 0.00 (0.00-0.00) | 0.705 |
| Anti-GP2 IgA | 0.00 (0.00-0.00) | 0.00 (0.00-0.00) | 0.583 |
| Anti-GP2 IgG | 0.00 (0.00-0.00) | 0.00 (0.00-0.00) | 0.845 |

Increased levels of Total secretory and ASCA IGA was associated with alcoholic etiology of liver cirrhosis. Data are presented as median (interquartile range). Ig: immunoglobulin; LC: light chain; sIgA: secretory IgA, EndoCab: endotoxin core antibody; ASCA: anti-*Saccharomyces* cerevisiae antibody; GP2: pancreatic glycoprotein 2.

**Supplementary Table 6. Association between alcoholic etiology and frequency of disease-related target-specific antibodies**

|  | | Non-alcoholic | Alcoholic | p |
| --- | --- | --- | --- | --- |
| ASCA IgA | Neg. | 14 (87.50%) | 52 (46.43%) | **0.002** |
|  | Pos. | 2 (12.50%) | 60 (53.57%) |  |
| ASCA IgG | Neg. | 12 (75.00%) | 93 (83.04%) | 0.486 |
|  | Pos. | 4 (25.00%) | 19 (16.96%) |  |
| Anti-F-actin IgA | Neg. | 9 (56.25%) | 34 (30.63%) | 0.052 |
|  | Pos. | 7 (43.75%) | 77 (69.37%) |  |
| Anti-F-actin IgG | Neg. | 10 (62.50%) | 70 (63.06%) | >0.999 |
|  | Pos. | 6 (37.50%) | 41 (36.94%) |  |
| Anti-Gliadin IgA | Neg. | 9 (56.25%) | 46 (41.44%) | 0.291 |
|  | Pos. | 7 (43.75%) | 65 (58.56%) |  |
| Anti-Gliadin IgG | Neg. | 14 (87.50%) | 93 (83.78%) | >0.999 |
|  | Pos. | 2 (12.50%) | 18 (16.22%) |  |
| Anti-GP2 IgA | Neg. | 13 (81.25%) | 84 (75.00%) | 0.760 |
|  | Pos. | 3 (18.75%) | 28 (25.00%) |  |
| Anti-GP2 IgG | Neg. | 15 (93.75%) | 104 (92.86%) | >0.999 |
|  | Pos. | 1 (6.25%) | 8 (7.14%) |  |

Increased frequency of ASCA IgA is observed in patients with alcoholic etiology of cirrhosis. Neg.: negative; Pos.: positive; Ig: immunoglobulin; ASCA: anti-*Saccharomyces* cerevisiae antibody; GP2: pancreatic glycoprotein 2.

**Supplementary Table 7. Correlation between number (#) of disease-related target-specific antibodies (ABs) and other immunoglobulin (Ig) markers**

|  | | total IgA | total IgG | EndoCab IgA | Kappa | Lambda |
| --- | --- | --- | --- | --- | --- | --- |
| #IgA ABs | r | 0.699 | 0.343 | 0.604 | 0.311 | 0.412 |
|  | p | **<0.001** | **<0.001** | **<0.001** | **<0.001** | **<0.001** |
| #IgG ABs | r | 0.322 | 0.428 | 0.334 | 0.190 | 0.200 |
|  | p | **<0.001** | **<0.001** | **<0.001** | **0.032** | **0.024** |

**Supplementary Table 8. Immunoglobulin A antibody levels are correlated with Child-Pugh score**

|  | | total IgA | sIgA | EndoCab IgA | ASCA IgA | Anti-F-actin IgA |
| --- | --- | --- | --- | --- | --- | --- |
| Child-Pugh score | r | 0.327 | 0.361 | 0.275 | 0.261 | 0.358 |
|  | p | **<0.001** | **<0.001** | **0.002** | **0.003** | **<0.001** |

**Supplementary Table 9. Clinical and laboratory characteristics of patients with acute-on-chronic liver failure (ACLF)**

|  | Discovery cohort (n=37) | Validation cohort (n=50) | p value |
| --- | --- | --- | --- |
| Age | 64 (56-70) | 61 (54-68) | 0.409 |
| Sex (m/f) | 27 / 10 (73% / 27%) | 27 / 23 (54% / 46%) | 0.079 |
| Etiology (alcohol / viral / both / other) | 31 / 1 / 3 / 2 (83.8% / 2.7% / 8.1% / 5.4%) | 26 / 9 / 2 / 13 (52.0% / 18.0% / 4.0% / 26.0%) | **0.004** |
| CLIF-C ACLF score | 49 (43-52) | 52 (45-59) | 0.062 |
| MELD score | 23 (19-30) | 24 (19-33) | 0.500 |
| White blood cells | 7.68 (5.75-10.18) | 10 (7.26-13.86) | **0.019** |
| Platelets | 134 (97-195) | 115 (71-169) | 0.336 |
| INR | 1.48 (1.16-2.10) | 1.45 (1.27-2.12) | 0.770 |
| Albumin (g/L) | 28 (23-32) | 27 (22-31) | 0.471 |
| AST (U/L) | 56 (28-105) | 68 (33-135) | 0.274 |
| ALT (U/L) | 28 (18-50) | 33 (21-75) | 0.253 |
| ALP (U/L) | 137 (92-221) | 117 (88-189) | 0.473 |
| γGT (U/L) | 105 (58-257) | 78 (47-169) | 0.287 |
| Bilirubin (µmol/L) | 51.2 (14.4-244.3) | 68.2 (35.4-260.4) | 0.115 |
| Creatinine (µmol/L) | 221 (113-286) | 194 (135-254) | 0.528 |
| CRP (mg/L) | 23 (9.53-72.20) | 39.1 (18.36-72.72) | 0.197 |
| mortality day-90 | 17 (45.9%) | 35 (70.0%) | **0.029** |

Data are presented as median (interquartile range) or n (%). CLIF-C: Chronic-Liver Failure Consortium; ACLF: acute-on-chronic liver failure; AD: acute decompensation; MELD: Model for End-Stage Liver Disease, INR: international normalized ratio; AST: aspartate aminotransferase, ALT: alanine aminotransferase, ALP: alkaline phosphatase, γGT: gamma-glutamyl transferase, CRP: C-reactive protein

**Supplementary Table 10. High serum sIgA levels according to both discovery and validation cohort cut-offs predicts 90-day mortality independently of ACLF grade in the merged ACLF cohort (n=87)**

| ACLF patients  (merged cohort)  90-day mortality | **Multivariable Cox regression analysis** | | |
| --- | --- | --- | --- |
|  | **HR** | **95% CI** | **p value** |
| ACLF 1 | reference | | |
| ACLF 2 | 3.429 | 1.520-7.737 | **0.003** |
| ACLF 3 | 6.708 | 2.768-16.260 | **<0.001** |
| sIgA (>20.9 µg/mL) | 2.309 | 1.020-5.228 | **0.045** |
| ACLF 1 | reference | | |
| ACLF 2 | 2.844 | 1.220-6.631 | **0.016** |
| ACLF 3 | 5.235 | 2.072-13.227 | **<0.001** |
| sIgA (>24.0 µg/mL) | 2.962 | 1.294-6.777 | **0.010** |

ACLF: acute-on-chronic liver failure; sIgA: secretory immunoglobulin A; HR: hazard ratio; CI: confidence interval.

**Supplementary Table 11. Liver, cerebral, circulatory and respiratory failures are associated with increased, while renal failure is associated with decreased serum secretory (s)IgA levels in patients with acute-on-chronic liver failure**

| Organ Failure | sIgA concentration (µg/mL) | | p |
| --- | --- | --- | --- |
|  | Absent | Present |  |
| Liver | 20.53 (11.92-38.15) | 58.93 (29.17-77.03) | **<0.0001** |
| Renal | 37.95 (24.64-62.7) | 23.15 (13.05-53.3) | **0.0435** |
| Cerebral | 25.34 (13.53-54.22) | 47.73 (23.61-72.99) | **0.0361** |
| Coagulation | 25.36 (14.25-57.54) | 35.45 (26.01-45.07) | 0.2631 |
| Circulatory | 22.7 (12.01-44.95) | 47.73 (27.25-69.3) | **0.0012** |
| Respiratory | 23.44 (13.53-50.81) | 43.07 (26.26-66.67) | **0.0341** |

Data are presented as median (interquartile range)

**Supplementary Table 12. No statistically significant difference was found in 90-day mortality rates between acute-on-chronic liver failure (ACLF) patients receiving and not receiving non-selective beta blockers (NSBBs)**

| Discovery cohort | | |
| --- | --- | --- |
| 90-day mortality | NSBB | |
|  | no | yes |
| Survived | 13 (50.0%) | 7 (63.6%) |
| Died | 13 (50.0%) | 4 (36.4%) |
| Fischer’s exact p = 0.495 | | |

**Supplementary Table 13. Secretory immunoglobulin A (sIgA) serum levels demonstrated excellent discriminatory capability for 90-day mortality in patients not receiving non-selective beta blockers (NSBBs) but could not differentiate between patients on this treatment**

| 90-day mortality | | Survivors* | Non-Survivors* | AUROC | 95%CI | p |
| --- | --- | --- | --- | --- | --- | --- |
| NSBB | no | 13.25  (7.25-19.39) | 39.40  (28.55-73.46) | 0.959 | (0.888-1.000) | **<0.001** |
|  | yes | 20.55  (13.80-44.95) | 26.60  (9.12-65.77) | 0.571 | (0.168-0.975) | 0.705 |

* median (interquartile range)
