# Supplementary material for: Secretory IgA Is a Key Marker Among Gut Barrier Dysfunction‐Related Immunoglobulins Predicting Outcomes in ACLF
Source: Liver Int. 2025 Sep 13;45(10):e70350. doi: 10.1111/liv.70350 (PMC12432679; doi:10.1111/liv.70350)
Supplement: Supplementary file 2 — Data S1: Supporting Information S1. [file LIV-45-0-s002.pdf]

BETEGTÁJÉKOZTATÓ

Vizsgálók által indított tudományos kutatás

Centrumszám: ☐☐ **Sorszám:** ☐☐☐ **Beteg nevének kezdőbetűi:** ☐☐☐

A vizsgálat címe: „PREDICT (Predicting Acute-on-Chronic Liver Failure in Cirrhosis) nemzetközi tanulmány" és ennek keretein belül a „Bél barrier károsodás szerepének vizsgálata a krónikus májbetegségre rakódott akut májelégtelenség kialakulásában" című kiegészítő tanulmány

A vizsgálat rövid címe: PREDICT

Kérjük, figyelmesen olvassa el az alábbi tájékoztatót!

Tisztelt Betegünk!

Ön kezelését végző belgyógyász/ gasztroenterológus szakorvos májzsugort és annak heveny rosszabbodását állapította meg és emiatt **vérvételt, vizelet és székletvizsgálatot** indikált.

Az Ön kezelőorvosa és az ellátásért felelős intézmény, a Debreceni Egyetem Klinikai Központ Belgyógyászati Intézete közösen egy nemzetközi, beavatkozással nem járó, megfigyeléses klinikai vizsgálatban vesz részt. **A vizsgálat célja,** hogy megértsük az Ön betegségének kialakulását, az ehhez vezető kóros folyamatokat, azok klinikai, szerológiai és genetikai hátterét. Kutatásaink során olyan információkhoz juthatunk, mely ezen betegség megállapításában és kezelésében jelentős fejlődéshez vezethetnek a jövőben. Vizsgálataink eredménye a későbbiekben csökkentheti a májzsugor heveny rosszabbodásában szenvedők kezelésének idejét vagy akár növelhetik a betegségek gyógyításának esélyeit.

Arra kérjük Önt, hogy vegyen részt a PREDICT című, illetve ezen nemzetközi vizsgálat fent említett, plusz beavatkozással nem járó kiegészítő tanulmányában.

A vizsgálat során az Ön kezelésének adatait szeretnénk **anonim módon** egy orvostudományi kutatásban felhasználni. **Kérjük, olvassa el figyelmesen az**

alábbiakat és amennyiben nincs ellenére, megkérnénk, hogy mintaadással járuljon hozzá kutatási erőfeszítéseinkhez, mellyel Önökön, betegeken kívánunk segíteni. Hozzájárulásáról nyilatkozhat a jelen dokumentum (ún. **Betegtájékoztató**), illetve ennek kiegészítő dokumentumának (ún. **Betegbeleegyező nyilatkozat**) aláírásával. Természetesen amennyiben Ön nem szeretne mintát adni, illetve részt venni a vizsgálatban, döntését tiszteletben tartjuk és megnyugtatjuk, hogy ez a további kezelésére, illetve az Önnel való bánásmódra semmilyen hatással sem lesz. Amennyiben a két dokumentummal kapcsolatban kérdése merülne fel, kérjük, forduljon bizalommal orvosához, aki ezeket a kérdéseket meg tudja az Ön számára is érthető módon válaszolni.

A betegtájékoztatást végző orvos:
Neve: ______________________________
Beosztása: ___________________________
Telefonszáma: _________________________

A vizsgálóhely adatai:
Neve: ____
Címe: ____

Koordináló vizsgálatvezető: **Dr. Papp Mária, PhD**
egyetemi docens
munkacsoportvezető
kutatásvezető
DEKK Belgyógyászati Intézet,
Gasztroenterológiai Tanszék
4032 Debrecen Nagyerdei krt. 98.
email: papp.maria@med.unideb.hu
tel.: +36-30 405-4993

A vizsgálat menete: Amennyiben Ön hozzájárul a vizsgálatban való részvételbe, **akkor sem kerül sor olyan orvosi beavatkozásra, vizsgálatra vagy soron kívüli ellenőrzésre, melyre egyébként a megfelelő ellátása érdekében nem lenne szükség.** A vizsgálatba való bevonás során ellenőrzik, hogy Ön alkalmas-e a vizsgálatba való részvételre. A vizsgálatban Európából vélhetőleg 1200 egyén vesz majd részt, akik májzsugorban szenvednek és annak heveny rosszabbodása miatt kórházi ellátást igényelnek. A vizsgálatot Magyarországon a Debreceni Egyetem Belgyógyászati Klinikája végzi.

Az **első orvosi vizsgálat** alkalmával, valamint a kórházi kezelés során és a májzsugor heveny szövődményéből való felépülést követően 1, 2 és **3 hónap múlva**, az **egyébként is esedékes orvosi felülvizsgálat** alkalmával szeretnénk az Ön betegségével kapcsolatos orvosi adatokat összegyűjteni. Ez magában foglalja az Ön általános állapotára vonatkozó adatokat és a szükséges orvosi kivizsgálás (betegvizsgálat,

laboratóriumi és képalkotó vizsgálatok), valamint a gyógyszeres és egyéb kezelések eredményeit. A betegség heveny szakának lezajlását követően is szeretnénk az Ön egészségének alakulását követni, így **egy éven keresztül háromhavonta** orvosi felülvizsgálatot tervezünk. Ez a vizsgálat nem foglal magában semmiféle speciális kezelést, csak az Ön orvosi adatainak összegyűjtését és áttekintését. A vizsgálatban való részvétel, beleértve az **1, 3, 6 és 12 hónap** múlva tervezett orvosi felülvizsgálatot, az Ön számára semmiféle időbeli többletterheléssel nem fog járni, mindössze az orvosi szóbeli tájékoztató meghallgatásával illetőleg az írásos betegtájékoztató figyelmes elolvasásával. A jelen vizsgálatban a betegkövetés teljes mértékben megegyezik a májzsugorban szenvedő betegek követésének klinikai gyakorlatával.

Az orvosi adatok összegyűjtése mellett mintavételeket is tervezünk. A betegektől történő mintavételt az Eü. M. 23/2002 rendelet 3.§.4. pontja ajánlását követve úgy terveztük, hogy a mintavétel Önnek **plusz orvosi beavatkozást ne** jelentsen.
Mintavétel: **Vérvétel**
A tudományos vizsgálat során vérvételre 3 alkalommal kerülne sor: az első orvosi vizsgálaton (1. nap), a kórházi bennfekvés során további egy alkalommal (7-10. nap), valamint a tervezett orvosi felülvizsgálatok során az első alkalommal (1 hónap), az egyébként is esedékes vérvételekkel egyidejűleg. Ez plusz szúrást Önnek így nem jelent, fájdalommal nem jár. Önre vonatkozóan semmilyen egészséget károsító hatása nincs. A tudományos vizsgálathoz 32 ml plusz vért vennénk le. Egy önkéntes véradás során az önkéntesek ennek a vérmennyiségnek sokszorosát adják problémamentesen.

A vérvételekkel egyidejűleg vizelet (5 ml), széklet egy hajszál minta adását is kérnénk. Továbbá amennyiben az Ön betegsége hasvízképződéssel (ascites) jár és a kezelőorvos hasvízcsapolást tart szükségesnek a kivizsgálás vagy a kezelés részeként, a hasvízből 10 ml minta eltételét tervezzük. Amennyiben az Ön betegsége hasvízképződéssel nem jár, vagy a kezelőorvos hasvízcsapolást nem tart szükségesnek, erre nem kerül sor.

Amennyiben az Ön kivizsgálása során felső és/ vagy alsó tápcsatornai endoszkópos vizsgálat válik indokolttá (pl. hasi panaszok, vérszékelés, vérhányás, székelteben rejtett vér megjelenése vagy jelentős vérszegénység miatt) és ennek során olyan elváltozást találunk, amelynek tisztázására szövettani mintavételre van szükség, illetőleg annak további vizsgálatára van szükség azt a Pathológiai Intézetbe továbbítjuk. A diagnosztikus beavatkozást megelőzően Önt az endoszkópiaval és szövettani vizsgálattal kapcsolatos tudnivalókról a mindennapi klinikai gyakorlatban használatos hivatalos betegtájékoztatóval és beleegyező nyilatkozattal ellátjuk. Jelenleg azt szeretnénk kérni, hogy az így nyert szövetmintákat a későbbiekben tovább vizsgálhassuk. A vizsgálatban a bél nyálkahártya működési zavarát szeretnénk elemezni és annak jelentőségét a májzsugor betegséglefolyásában.

Amennyiben a fent részletezett tünetek nem állnak fenn és így a kezelőorvos endoszkópos vizsgálatot nem tart szükségesnek, erre nem kerül sor.

Kutatás menete és az adatkezelés módja:
Az orvosi adatok és a minták minden esetben azonnal egy **kódszám**ot kapnak, így az **Ön neve végig titkosan** lesz kezelve, azt csak kezelőorvosa és a kutatást végző orvos ismeri. Az **adatok és a minták** (vér, vizelet, széklet, nyál, hasvíz és bélbiopsziás minták) **tárolása** a kutatás elvégzéséig biobankban (Biobank HCB-IDIBAPS (Anna Bosch-Comas, PhD. IDIBAPS BIOBANK, Email: abosch1@clinic.ub.es, Phone: +34 93 227 5707 ext. 4303 or +34 93 227 5400 ext. 4303) történik. A minták vizsgálatára egyrészt a Debreceni Egyetem Klinikai Központ Belgyógyászati Intézetének laboratóriumában, illetőleg a vizsgálat nemzetközi volta miatt külföldi kutatólaboratóriumokban kerül sor. A vizsgálatokat követően, ha a minták nem kerülnek teljes egészében felhasználásra szeretnénk azokat jövőbeli kutatások céljából eltárolni, és amennyiben a betegség kórlefolyásával kapcsolatosan új szempontok vagy vizsgálómódszerek látnak napvilágot ismételten megvizsgálni. A jelen vizsgálatokból nyert eredmények valószínűleg az Ön kezelésére már nem lesznek hatással, azonban a későbbiekben a betegségek gyógyítását elősegíthetik. A mintákból genetikai vizsgálatot is végzünk. Az adatokat és kódkulcsokat mind fizikailag és elektronikusan elkülönítve tároljuk. A személyazonosító adatokkal együttesen tárolt, valamint kódolt genetikai mintát illetve adatot tartalmazó nyilvántartás személyazonosító adatokat tartalmazó nyilvántartással nem kerül összekapcsolásra.

A vizsgálat időtartama és vizsgálatba való beleegyezés visszavonhatósága: A biobank a tárolt minden adatot és mintát, valamint az ezekkel kapcsolatos minden eljárást, tevékenységet 2030. december 31-ig tart nyilván, kivéve, ha az érintett az adat és mintakezeléséhez adott beleegyezését visszavonja. Visszavonás esetén az érintett tájékoztatását követően valamennyi az adatokra vonatkozó nyilvántartást megsemmisítik.

Előnyök és hátrányok ismertetése: A kutatásban való részvétel az Ön egészségi állapotát nem befolyásolja, Önnek a részvételből sem hátránya sem előnye nem származik. A vizsgálatban való részvétel teljesen önkéntes, nem jár fájdalommal, Önnek és többlet költséggel sem.

Díjazás: A kutatásban való részvételért Ön semmilyen fizetségben, költségtérítésben vagy egyéb juttatásban nem részesül. Az Ön által biztosított biológiai minták jövőbeli kutatásban való felhasználása olyan új termékeket, teszteket vagy felfedezéseket eredményez, melyek potenciális kereskedelmi értékkel bírhatnak. A mintaadónak mintára vonatkozó tulajdonjoga nincs. Mint ilyen, nem osztozik a termékekből, tesztekből vagy felfedezésekből származó pénzügyi haszonból.

A vizsgálatba való beleegyezés önkéntes és befolyásolástól mentes, azt bármikor akár szóban, akár írásban indokolás nélkül vissza lehet vonni anélkül, hogy ebből Önnek hátránya származna.

Cím: H-4032 Debrecen, Nagyerdei krt. 98. ¤ Tel.: 36-52-255-525 ¤ Fax: 36-52-255-951

Kockázatok és esetleges kár enyhítésének módja: Tekintettel arra, hogy a vizsgálatok során a beteg kezelést nem kap, illetve nem történik olyan vizsgálat, ami a belgyógyászati/ gasztroenterológiai szakterületen ne lenne már ismert, **a mintavételnek specifikus kockázata nincs**. A vizsgálatok hasznosak, hiszen a klinikai adatok összegyűjtésével, valamint a szerológiai és genetikai vizsgálatok eredményei által megismerhetjük a májzsugor heveny rosszabbodásának patofiziológiai folyamatait, ami később diagnosztikus, illetve terápiás megoldásokhoz vezethet. A kezelőszemélyzet a megfelelő munkavédelmi előírások betartásával dolgozik.

Engedélyek: A vizsgálatot Magyarországon az Egészségügyi Tudományos Tanács – Tudományos és Kutatásetikai Bizottság (ETT-TUKEB), mint felelős magyar hatóság engedélyezte. A vizsgálatot a vonatkozó hazai jogszabályok és a nemzetközi irányelvek szigorú betartásával folytatjuk.

A mintákon folytatott vizsgálatok eredményeit kizárólag kutatási célokra használják fel, a tesztek eredményeit nem kapja meg a mintát biztosító beteg.

Amennyiben az említett vizsgálatokkal kapcsolatban kérdése lenne, úgy kérem forduljon kezelőorvosához bizalommal. A későbbiekben, ha szeretné tudni a vizsgálatok menetét és a kutatási projekt előrehaladását, úgy érdeklődjön Dr. Papp Mária kutatásvezetőnél (elérhetőség l.d. fent) vagy kezelőorvosánál.

Aláírás előtt és a vizsgálat alatt kezelőorvosától bármikor további tájékoztatást kérhet:

Az orvos neve (nyomtatott betűkkel): ______________________

Beosztása: ______________________

Az orvos aláírása ______________________

A tájékoztatás dátuma (sajátkezűleg) ______________________

Aláírásommal kijelentem, hogy jelen tájékoztatót elolvastam és annak egy eredeti példányát átvettem:

A beteg neve (nyomtatott betűkkel) [1]: ______________________

A beteg aláírása: ______________________

Aláírás dátuma (sajátkezűleg) ______________________

Dátum ______________________

---

[1] Korlátozottan cselekvőképes vagy cselekvőképtelen résztvevők esetén a törvényes képviselő tölti ki

Aláírásommal kijelentem, hogy a beteg törvényes képviselőjeként jelen tájékoztatót elolvastam és annak egy eredeti példányát átvettem:

A törvényes képviselő neve *(nyomtatott betűkkel)* [1]: ______________________

A törvényes képviselő aláírása: ______________________

Aláírás dátuma *(sajátkezűleg)* ______________________

Anyja neve: ______________________

Születési hely és idő: ______________________

Szem. ig. szám: ______________________

Lakcím: ______________________

Dátum ______________________

Köszönjük, hogy időt szánt e tájékoztató végigolvasására. Ha úgy döntött, hogy részt vesz a vizsgálatban, kérjük, írja alá a csatolt Beleegyezési nyilatkozatot is.

BELEEGYEZŐ NYILATKOZAT

1. A kutatás azonosító adatai:

A vizsgálat címe**: „PREDICT (Predicting Acute-on-Chronic Liver Failure in Cirrhosis) nemzetközi tanulmány**” illetőleg az ehhez kapcsolódó „**Bél barrier károsodás szerepének vizsgálata a krónikus májbetegségre rakódott akut májelégtelenség kialakulásában**” kiegészítő tanulmány

Obszervációs prospektív klinikai vizsgálat

A kérelmező neve, munkaköre és beosztása: Dr. Papp Mária, belgyógyász-gasztroenterológus, egyetemi docens

2. Egészségügyi intézmény megnevezése

Debreceni Egyetem, Klinikai Központ, Belgyógyászati Intézet

3. A kutatás vezetőjének, illetve a tájékoztatást adónak a neve, beosztása, munkaköre

A kutatás vezetője: Dr. Papp Mária

Munkahely: Debreceni Egyetem, Klinikai Központ, Belgyógyászati Intézet, Gasztroenterológiai Tanszék

Beosztása/munkakör: egyetemi docens

A tájékoztatást végző személy:……………………………………………………..

Munkahely: Debreceni Egyetem, Klinikai Központ, Belgyógyászati Intézet

Beosztása/munkakör:………………………………………………………………..

4. A résztvevő adatai

NÉV: ……………………………………………………………………………………..

Lakcím: ……………………………………………………………………………......

Anyja neve:………………………………………………………………………………

Születési hely és idő: ……………………………………………………………………

TAJ szám:……..-………-……….

Alulírott a fenti vizsgálatban való részvételemet vállalom, abba beleegyezem.

1. Elolvastam és megértettem a tájékoztató adatlapot, lehetőségem volt kérdéseket feltenni és azokra mindenben kielégítő választ kaptam.
2. Tudomásul veszem, hogy a vizsgálatban önként, szabad elhatározásomból, befolyástól mentesen veszek részt, bármikor szabadon, indoklás nélkül, szóban vagy írásban visszaléphetek és ez sem a kezelésemet, sem a jogaimat nem fogja befolyásolni.
3. Tudomásul veszem, hogy a rám vonatkozó anonimizált orvosi feljegyzések egy részébe a vizsgálatban résztvevő személyek betekintenek, ehhez hozzájárulok.
4. Beleegyezem, hogy személyes adataimat nem tartalmazó, a vizsgálat során gyűjtött adataimat és biológiai mintáimat (vér, haj, széklet, vizelet, nyál esetlegesen hasvíz, bélbiopsziás minta) elemzésekre felhasználják, azokat orvosi szaklapban közöljék.
5. Beleegyezem, hogy biológiai mintáimat (vér, haj, széklet, vizelet, nyál esetlegesen hasvíz, bélbiopsziás minta) anonim módon, biobankban tárolják és későbbi vizsgálatokhoz felhasználják.
6. Beleegyezem, hogy a levett biológiai mintáimat jövőbeli kutatásokhoz felhasználják, és ígérem, hogy a kutatáshoz biztosítom a Minimálisan Szükséges Adatokat, melyek személyazonossági információkat nem tartalmaznak. Ezek az információk a mikrobióma elemzés során kerülnek felhasználásra, és felhasználhatóak tudományos publikációkban valamint nyomon követéses kutatásokban. A személyes kilétem azonban mindvégig anonim és bizalmas marad.
7. Beleegyezem, hogy a biológiai mintáimhoz kapcsolódó adatokat (beleértve a mikrobiómára vonatkozó adatokat is) a Minimálisan Szükséges Adatokkal egyetemben nyilvánosságra hozzák. A titkosított adatok és a biológiai minták kérésre más, a PREDICT-en belüli orvosi-kutatási projektekre, valamint külső fél számára is átadhatók. Ezek a felek lehetnek hazai vagy külföldi kutatóintézetek vagy ipari partnerek. A harmadik félnek továbbított biológiai mintákat és adatokat kizárólag alkalmazott kutatási projektekhez szabad felhasználni, azokat további felek részére továbbadni szigorúan tilos.

_______________	__________________________
dátum	a beteg aláírása
_______________	__________________________
dátum	a felvilágosítást végző orvos aláírása

....

[1] Korlátozottan cselekvőképes vagy cselekvőképtelen résztvevők esetén a törvényes képviselő tölti ki

Aláírásommal kijelentem, hogy a beteg törvényes képviselőjeként jelen tájékoztatót elolvastam és annak egy eredeti példányát átvettem:

A törvényes képviselő neve *(nyomtatott betűkkel)*[1]: ________________________________

A törvényes képviselő aláírása: ________________________________

Aláírás dátuma *(sajátkezűleg)* ________________________________

Anyja neve: ________________________________

Születési hely és idő: ________________________________

Szem. ig. szám: ________________________________

Lakcím: ________________________________

DEBRECENI EGYETEM
ÁLTALÁNOS ORVOSTUDOMÁNYI KAR
BELGYÓGYÁSZATI INTÉZET
GASZTROENTEROLÓGIAI TANSZÉK
TANSZÉKVEZETŐ: DR. ALTORJAY ISTVÁN EGYETEMI TANÁR

VISSZALÉPÉSI NYILATKOZAT

Alulírott,…………………………………… kérem, hogy a **„PREDICT (Predicting Acute-on-Chronic Liver Failure in Cirrhosis) nemzetközi tanulmány" és ennek keretein belül a „Bél barrier károsodás szerepének vizsgálata a krónikus májbetegségre rakódott akut májelégtelenség kialakulásában" című kiegészítő tanulmány"** –ba bekerült adataimat töröljék, illetve a levett biológiai mintáimat megsemmisítsék. A kutatásban a továbbiakban nem kívánok részt venni. Visszalépésem után semmilyen kötelezettséget a tanulmányt végző intézettel illetve kutatókkal szemben nem támasztok.

Dátum: **…………**

…………………………..
Beteg aláírása

Cím: H-4032 Debrecen, Nagyerdei krt. 98. ¤ Tel.: 36-52-255-525 ¤ Fax: 36-52-255-951
